# A MYC family switch: L-MYC drives and maintains neuroendocrine lineage programs in prostate cancer

**DOI:** 10.1016/j.neo.2026.101307

**Published:** 2026-04-17

**Authors:** Jeyaluxmy Sivalingam, Kaleigh Ballagh, Kyung Hyun Cho, Yingli Shi, Lin Li, Preston Barron, Michael S. Lan, Omar E Franco, Xiuping Yu

**Affiliations:** a Department of Biochemistry & Molecular Biology, LSU Health-Shreveport, LA, USA; b School of Medicine, LSU Health-Shreveport, LA, USA; c Department of Genetics, LSU Health-New Orleans, LA, USA; d Department of Urology, LSU Health-Shreveport, LA, USA

**Keywords:** Prostate cancer, Neuroendocrine, MYCL

## Abstract

Neuroendocrine prostate cancer (NEPC) is an aggressive, therapy-resistant subtype that emerges through lineage plasticity following androgen receptor pathway inhibition. Although MYC family oncogenes are central to prostate cancer progression, the role of MYCL (L-MYC) in NEPC has remained poorly defined. Here, we show that MYCL is selectively and robustly upregulated in NEPC patient samples and experimental models, whereas MYC is concurrently suppressed and MYCN remains low, revealing a lineage-associated MYC family switch. MYCL expression strongly correlates with the neuroendocrine lineage regulators ASCL1 and INSM1 and inversely with adenocarcinoma-associated genes. Functionally, MYCL overexpression suppresses androgen receptor signaling, induces neuroendocrine-like transcriptional reprogramming, and remodels cytoskeletal and adhesion pathways associated with cellular plasticity, whereas MYCL knockdown disrupts neuroendocrine lineage identity and restores adenocarcinoma-associated gene expression, including MYC. Mechanistically, MYC suppression is associated with transcriptional regulation by ASCL1, while MYCL upregulation appears to occur within a permissive epigenetic landscape rather than through genomic amplification. Together, these findings identify MYCL as a lineage-specific regulator that drives and maintains neuroendocrine identity and define a MYC family regulatory switch in which MYCL replaces MYC to stabilize neuroendocrine lineage programs in advanced prostate cancer.

## Highlights

• First report of MYCL expression and function in NEPC.
• MYCL up and MYC down, lineage-associated MYC switch in PCa.
• MYCL activation reflects a permissive epigenetic state.
• MYCL suppresses AR and drives neuroendocrine-like reprogramming.
• MYCL depletion restores adenocarcinoma genes, including MYC.

## Introduction

Prostate cancer (PCa) is the most commonly diagnosed non-skin cancer and the second leading cause of cancer-related deaths in U.S. men [1]. Early stage PCa is typically androgen-dependent and responds to androgen deprivation therapy; however, over time, most tumors eventually progress to castration-resistant PCa [2]. While many castrate-resistant PCa cases continue to respond to AR pathway inhibitors, a subset of tumors becomes resistant through lineage plasticity, leading to the emergence of neuroendocrine prostate cancer (NEPC) [3]. The incidence of NEPC has increased since the widespread clinical use of next generation of AR pathway inhibitors around 2012. Notably, NEPC and adenocarcinoma prostate cancer (AdPC) share a largely conserved genomic landscape but exhibit distinct epigenomic signatures [3]. During this phenotypic shift, AR expression is lost and neuroendocrine (NE) lineage markers such as ASCL1, INSM1 and CHGA are upregulated [3]. Currently, there are no effective treatments for NEPC, and the disease is associated with a poor prognosis, with a median overall survival of approximately 9 months [4]. Therefore, a deeper understanding of the molecular mechanisms driving the development of NEPC continues to be a priority for advancing PCa research.

The Myelocytomatosis (MYC) family of oncogenes, comprising MYC (c-MYC), MYCN (N-MYC), and MYCL (L-MYC), encodes transcription factors that regulate approximately 15% of all genes, including those involved in stress responses, proliferation, differentiation, cell cycle progression, apoptosis, and immune regulation [5]. The dysregulation of MYC family members has been implicated in tumorigenesis across multiple cancer types, including PCa. In AdPC, MYC is frequently amplified and overexpressed, where it promotes tumor progression [6,7]. In contrast, MYCN amplification is observed in approximately 40% of NEPC cases [8]. Notably, MYCN, in cooperation with activated AKT1, can transform human prostate epithelial cells and drive NEPC progression [9]. Additionally, MYCN can recruit EZH2 to suppress AR signaling and promote NE features [10].

While MYC and MYCN have been extensively studied in PCa, MYCL has primarily been described only at the level of focal copy-number amplification in clinically localized PCa [11]. Its regulation and functional role remain poorly understood, especially in NEPC, where MYCL is expressed [12].

Our analysis of publicly available patient and cell-line RNA-seq datasets revealed that MYCL expression is selectively upregulated in NEPC, whereas MYC is preferentially enriched in AdPC, while MYCN expression remains comparatively low in NEPC. This reciprocal expression pattern between MYCL and MYC suggests the existence of a MYC family switch associated with lineage plasticity and NE transdifferentiation. Here, we report that MYCL expression is markedly elevated in NEPC and provide mechanistic insights into its function in PCa, uncovering its epigenetic regulation and a previously unrecognized role in maintaining NE lineage programs. Collectively, our findings identify MYCL as a key transcriptional regulator that supports NE identity in NEPC.

## Results

### MYCL is upregulated and MYC is downregulated in NEPC across patient samples and cell line models

**Fig. 1:**
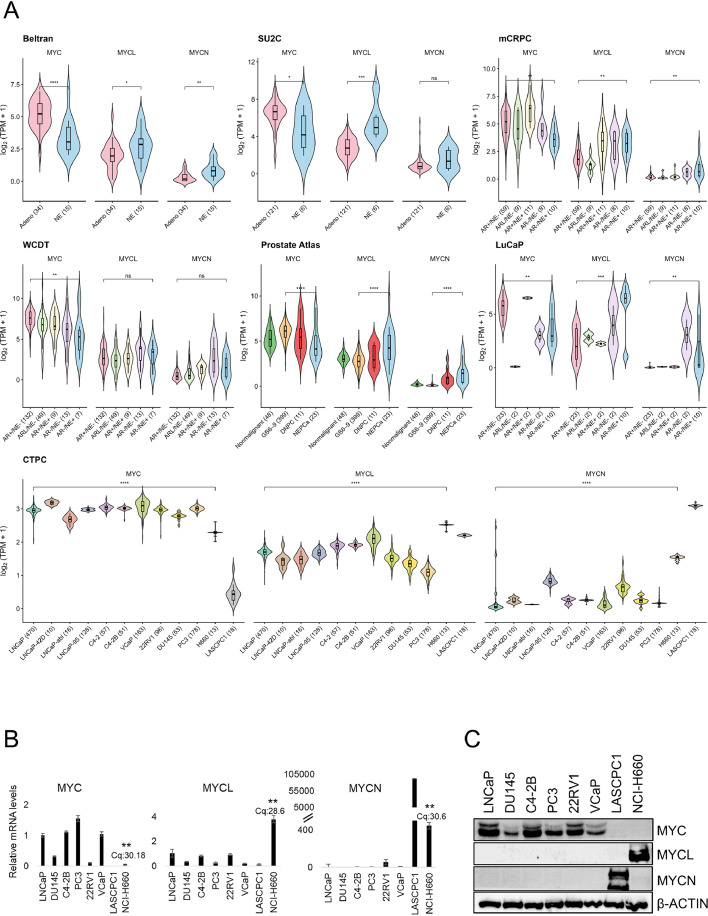
MYCL is upregulated and MYC is downregulated in neuroendocrine prostate cancer (A) Violin plots showing normalized mRNA expression levels of MYC, MYCL, and MYCN across multiple prostate cancer patient cohorts and experimental models, including the Beltran, SU2C, Labrecque mCRPC, WCDT, ProAtlas, LuCaP patient-derived xenograft (PDX), and CTPC datasets. Gene expression values are presented as log₂ (TPM + 1). Samples are grouped according to molecular subtype or lineage state, including AR⁺/NE⁻, AR⁺/NE⁺, AR-low/NE⁻ (ARL/NE⁻), AR⁻/NE⁻, and AR⁻/NE⁺, based on previously defined molecular classifications or adenocarcinoma versus neuroendocrine prostate cancer annotations. Across datasets, MYC expression predominates in adenocarcinoma tumors, whereas MYCL expression is enriched in neuroendocrine prostate cancer. MYCN expression remained comparatively low overall. (B) Quantitative RT-PCR analysis of relative mRNA expression levels of MYC, MYCL, and MYCN across prostate cancer cell lines. Expression levels were normalized to GAPDH and are presented relative to the reference cell line LNCaP. The neuroendocrine prostate cancer cell line NCI-H660 exhibits elevated MYCL, whereas adenocarcinoma models predominantly express MYC. Compared to other prostate cancer cell lines (excluding LASCPC1), MYC is significantly reduced and MYCL is significantly increased in H660. (C) Immunoblot analysis confirming protein expression of MYC family members in representative prostate cancer cell lines. β-ACTIN serves as a loading control. Consistent with transcriptomic findings, MYC protein is broadly expressed in adenocarcinoma models, whereas MYCL is preferentially detected in neuroendocrine prostate cancer cells.

Transcriptomic analysis across multiple PCa patient cohorts, including Beltran, SU2C, Labrecque mCRPC cohort, West Coast Prostate Cancer Dream Team (WCDT), and ProAtlas datasets, together with patient-derived xenograft models and PCa cell-lines (LuCaPs and CTPC), revealed lineage-associated divergence in MYC family gene expression during disease progression (Fig. 1A). MYC expression predominated in adenocarcinoma tumors but was markedly reduced in NEPC, with MYC protein undetectable in the NCI-H660 NEPC model. In contrast, MYCL was consistently and selectively upregulated in NEPC patient samples and exhibited strong enrichment at both transcript and protein levels. MYCN expression was detected in some LuCaP tumors but overall remained comparatively low across patient cohorts and was not detected at the protein level in NCI-H660 NEPC model (Fig. 1A, B, C).

Data are presented as mean ± SEM. Statistical significance was determined using a two-tailed Wilcoxon rank-sum test (*P < 0.05, **P < 0.01, ***P < 0.001, ****P < 0.0001).

Single-cell RNA-seq analysis further demonstrated that MYCL was enriched in NEPC populations as well as AR-high adenocarcinoma (AdPC_ARhi) cells, a distinct castration-resistant state, suggesting that MYCL activation may occur across multiple resistant lineages (Supplementary Fig. 1). Elevated MYCL expression closely correlated with canonical NEPC markers, including ASCL1, INSM1, and CHGA (Supplementary Fig. 2, 3), whereas MYC expression displayed an inverse association with NE programs and instead aligned with adenocarcinoma-associated genes such as NKX3-1, YAP1, and KLK3. Notably, relatively high MYCL and NEPC marker mRNA expression was also observed in VCaP cells, an amphicrine PCa model exhibiting both adenocarcinoma and NE features, supporting a potential role for MYCL in transitional lineage states bridging adenocarcinoma and NE phenotypes (Fig. 1A).

RT-qPCR and immunoblotting confirmed elevated MYCL mRNA and protein expression in NCI-H660 cells compared with adenocarcinoma models, whereas MYC transcript levels were reduced and MYC protein remained absent (Fig. 1B, C). Although MYCN mRNA exhibited apparent fold enrichment, its absolute transcript abundance remained low (as reflected by high Cq values) and MYCN protein was not detectable across the models examined. Collectively, these findings identify MYCL as a NE lineage-associated MYC family member in advanced PCa and support a model in which reciprocal suppression of MYC accompanies MYCL activation, consistent with a MYC family switch underlying NE transdifferentiation.

### MYCL has limited effects on proliferation but promotes migration and cytoskeletal remodeling in prostate cancer cells

**Fig. 2:**
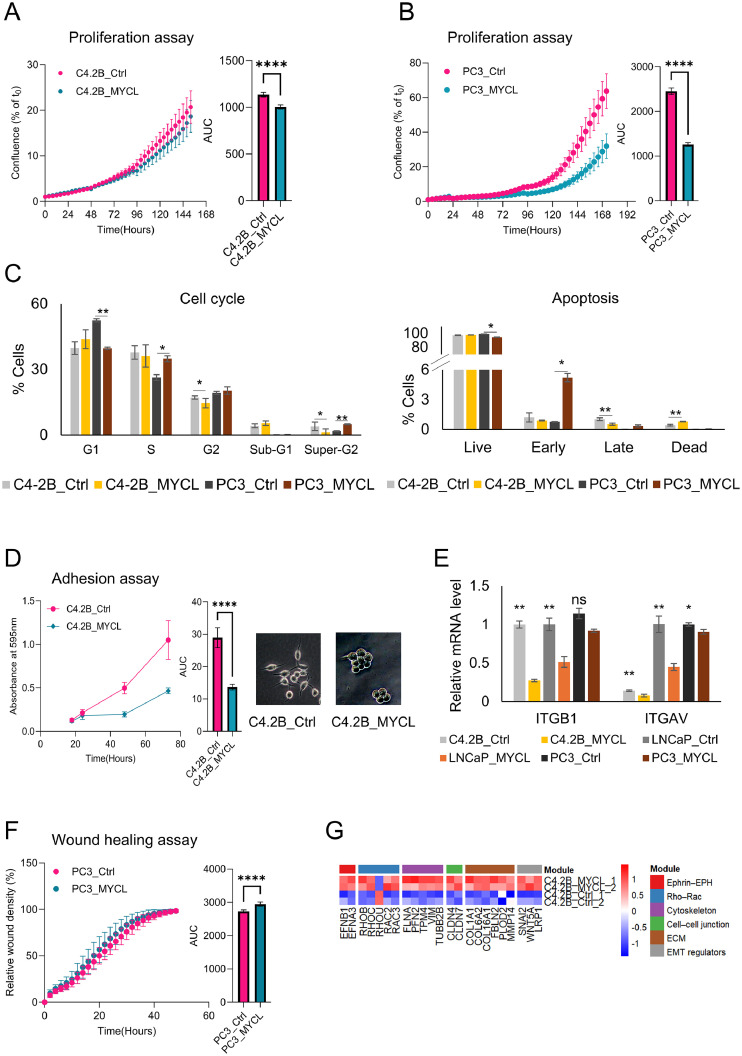
MYCL reduces proliferation while promoting migration and cytoskeletal remodeling and decreasing cell adhesion in prostate cancer cells (A) Proliferation assay. Real-time Incucyte analysis showing cell proliferation of C4-2B control (Ctrl) and C4-2B-MYCL cells measured as confluence (%) over time. Quantification using area under the curve (AUC) demonstrates reduced proliferative capacity following MYCL overexpression. (B) Proliferation assay. Real-time Incucyte analysis showing cell proliferation of PC3 control (Ctrl) and PC3-MYCL cells measured as confluence (%) over time. Quantification using area under the curve (AUC) demonstrates reduced proliferative capacity following MYCL overexpression. (C) Left: Cell cycle analysis of C4-2B and PC3 cells following MYCL overexpression. In PC3 cells, MYCL reduces the G1 population and increases S-phase, indicating altered cell cycle progression, whereas changes in C4-2B cells are minimal. Right: Apoptosis analysis by Annexin V/7-AAD staining. MYCL overexpression in PC3 cells increases the early apoptotic population, with little or no significant change in C4-2B cells. (D) Cell adhesion assay. Cell adhesion was assessed by crystal violet staining and quantified by measuring absorbance at 595 nm at 18, 24, 48, and 72 h following seeding. MYCL-overexpressing C4-2B cells exhibited significantly decreased adhesion compared with control cells. Representative phase-contrast images acquired 48 h after seeding show reduced cell attachment and increased cell clustering in MYCL-expressing cells. (E) Molecular validation. RT-qPCR analysis of adhesion-related genes (ITGB1, ITGAV) in control (Ctrl) and MYCL-overexpressing C4-2B, LNCaP and PC3 cells. (F) Migration assay. Wound-healing analysis measuring relative wound density (%) over time demonstrates moderately enhanced migratory capacity in PC3-MYCL cells. AUC quantification confirms increased migration upon MYCL expression. (G) Cytoskeletal transcriptional programs. Heatmap showing differential expression of genes meeting thresholds of |log₂FC| ≥ 0.5 and adjusted p-value (FDR) < 0.05. Differentially regulated genes are associated with Ephrin-EPH signaling, Rho-Rac signaling, cytoskeletal organization, cell-cell junctions, extracellular matrix (ECM) interactions, and epithelial-mesenchymal transition (EMT) regulators, indicating MYCL-driven cytoskeletal remodeling signatures.

MYCL overexpression in AdPC cells resulted in a mild reduction in proliferative capacity, as determined by both cell counting and IncuCyte live-cell imaging, with only modest differences in overall growth kinetics between control and MYCL-overexpressing C4-2B and PC3 cells (Fig. 2A, B; Supplementary Fig. 4A, B). Consistent with this, cell-cycle analysis revealed a reduction in the G1 population and a corresponding increase in S-phase in PC3 cells, accompanied by a modest increase in apoptosis, resulting in a slight decrease in net cell accumulation. In contrast, cell-cycle analysis showed a moderate increase in the G1 population in C4-2B/MYCL cells compared with controls; however, this difference did not reach statistical significance (p = 0.08). Apoptosis analysis revealed no significant change in early apoptosis, while late apoptosis was marginally reduced and the proportion of dead cells was slightly increased (Fig. 2C). Collectively, these results indicate that MYCL overexpression has minimal impact on cell-cycle distribution and apoptosis, suggesting that MYCL may not primarily regulate proliferation in C4-2B cells but may instead contribute to alternative oncogenic processes.

*Data are presented as mean ± SEM. Statistical significance was determined using a two-tailed Student’s test (*P < 0.05, **P < 0.01, ***P < 0.001, ****P < 0.0001).

Interestingly, MYCL markedly impaired cell adhesion, with MYCL-overexpressing cells displaying reduced attachment efficiency and altered cellular morphology in adhesion assays (Fig. 2D). Live-cell imaging further revealed defective cell spreading and persistent attachment failure following seeding, characterized by rounded and weakly adherent cells, characteristics often observed in NE tumors (Supplementary Video). Consistent with these observations, RT-qPCR analysis confirmed reduced expression of integrins (ITGB1 and ITGAV) in C4-2B, LNCaP, and PC3 cells (Fig. 2E). The coordinated downregulation of integrins critical for extracellular matrix engagement, particularly ITGB1, suggests impaired adhesion-dependent signaling.

Wound-healing assays demonstrated a mild but significant increase in migratory behavior in MYCL-overexpressing PC3 cells (Fig. 2F). Transcriptomic profiling revealed extensive MYCL-dependent reprogramming of cytoskeletal regulatory networks associated with cellular plasticity (Fig. 2G). Differential expression analysis identified significant alterations in genes governing Ephrin-EPH signaling, Rho-Rac GTPase activity, focal adhesion dynamics, and extracellular matrix remodeling, supporting cytoskeletal reorganization and increased cellular motility.

Collectively, these findings indicate that MYCL primarily drives cytoskeletal and adhesion remodeling rather than proliferative expansion, promoting a phenotypic transition toward increased cellular plasticity consistent with NE differentiation.

### MYCL drives neuroendocrine-like transcriptional reprogramming and suppresses MYC signaling

**Fig. 3:**
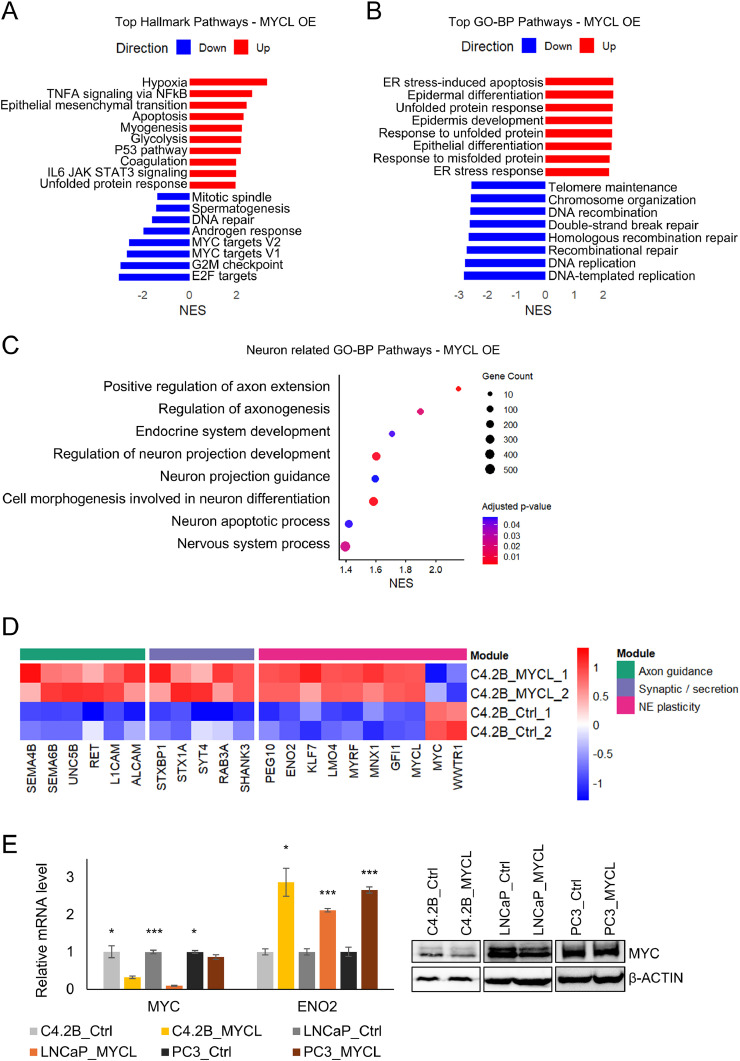
MYCL drives neuroendocrine-like transcriptional reprogramming and suppresses MYC signaling (A) Hallmark pathway enrichment analysis. GSEA of MSigDB Hallmark pathways in MYCL-overexpressing C4-2B cells versus controls (FDR < 0.05). Positively enriched pathways (red) include hypoxia, epithelial-mesenchymal transition, inflammatory signaling, and metabolic programs, whereas negatively enriched pathways (blue) include MYC target genes, G2/M checkpoint, DNA repair, and mitotic spindle processes. (B) Gene Ontology Biological Process (GO-BP) enrichment analysis following MYCL overexpression (FDR < 0.05). Enriched pathways indicate activation of stress response, metabolic adaptation, and differentiation-associated programs, with repression of DNA replication, DNA repair, and chromosome organization. (C) Neuron-associated GO-BP enrichment analysis. Neuronal pathways were filtered using FDR < 0.05 and reduced by semantic similarity clustering (similarity cutoff = 0.7). Dot size represents gene count per pathway, and color indicates adjusted P value (red = lower FDR; blue = higher FDR). MYCL overexpression enriches transcriptional programs related to axon extension, neuron projection development, and neuronal differentiation. (D) Neuroendocrine and axon-guidance transcriptional modules. Heatmap showing log2(TPM + 1) expression of genes involved in axon guidance, synaptic/secretion pathways, and neuroendocrine plasticity modules in control and MYCL-overexpressing cells, demonstrating activation of neuronal lineage programs. Color scale represents relative expression from low (blue) to high (red). Genes were selected using differential expression thresholds of |log2FC| ≥ 0.4 and FDR < 0.05. (E) Molecular validation of MYCL-driven transcriptional changes. RT-qPCR analysis confirms altered expression of MYC and ENO2 mRNA levels in MYCL-overexpressing C4-2B, LNCaP and PC3 cells relative to controls. Immunoblot analysis validates MYC protein expression with β-ACTIN as a loading control.

Transcriptomic profiling revealed that MYCL overexpression induces broad transcriptional reprogramming in PCa cells. Gene set enrichment analysis of Hallmark pathways demonstrated enrichment of hypoxia, epithelial-mesenchymal transition, and glycolysis programs, accompanied by suppression of androgen response and MYC target gene signatures in MYCL-overexpressing cells (Fig. 3A). Gene Ontology analysis further revealed upregulation of multiple differentiation-associated biological processes together with coordinated downregulation of pathways involved in DNA replication, DNA repair, and chromosome organization, indicating attenuation of proliferative and genome maintenance programs (Fig. 3B).

*Data are presented as mean ± SEM. Statistical significance was determined using a two-tailed Student’s test (*P < 0.05, **P < 0.01, ***P < 0.001, ****P < 0.0001).

Notably, neuronal and axon-related gene programs-including regulation of axonogenesis, neuron projection development, cell morphogenesis involved in neuron differentiation, nervous system processes and endocrine system development-were significantly enriched following MYCL overexpression (Fig. 3C). Consistent with these findings, heatmap analysis demonstrated coordinated upregulation of genes associated with axon guidance, synaptic and secretory pathways, and NE plasticity in MYCL-overexpressing C4-2B cells compared with control cells (Fig. 3D). Notably, key axon guidance regulators, including L1CAM, RET, and UNC5B were significantly increased following MYCL overexpression. Genes involved in synaptic and secretory function, such as STXBP1 and SYT1, were also upregulated, indicating activation of neurosecretory programs. Importantly, canonical NE plasticity markers, including PEG10 and ENO2, showed strong induction with elevated MYCL expression. Collectively, these findings suggest that MYCL drives activation of neuronal and NE transcriptional networks associated with lineage plasticity in PCa cells.

RT-qPCR validation confirmed reduced MYC expression in MYCL-overexpression cells, accompanied by increased expression of the NEPC marker ENO2. Consistently, immunoblot analysis demonstrated suppression of MYC protein levels in LNCaP and C4-2B cells, but not in PC3 cells, likely due to MYC gene amplification in this line (Fig. 3E, Supplementary Fig. 5B). Together, these findings support a MYC family switch in which MYCL activation coincides with repression of MYC signaling and induction of NE-associated transcriptional programs.

Collectively, these findings identify MYCL as a key regulator of MYC family switching that enables transcriptional reprogramming from an androgen receptor-dependent adenocarcinoma state toward a NE lineage program.

### MYCL suppresses androgen receptor signaling and promotes reduced dependence on AR pathway activity

**Fig. 4:**
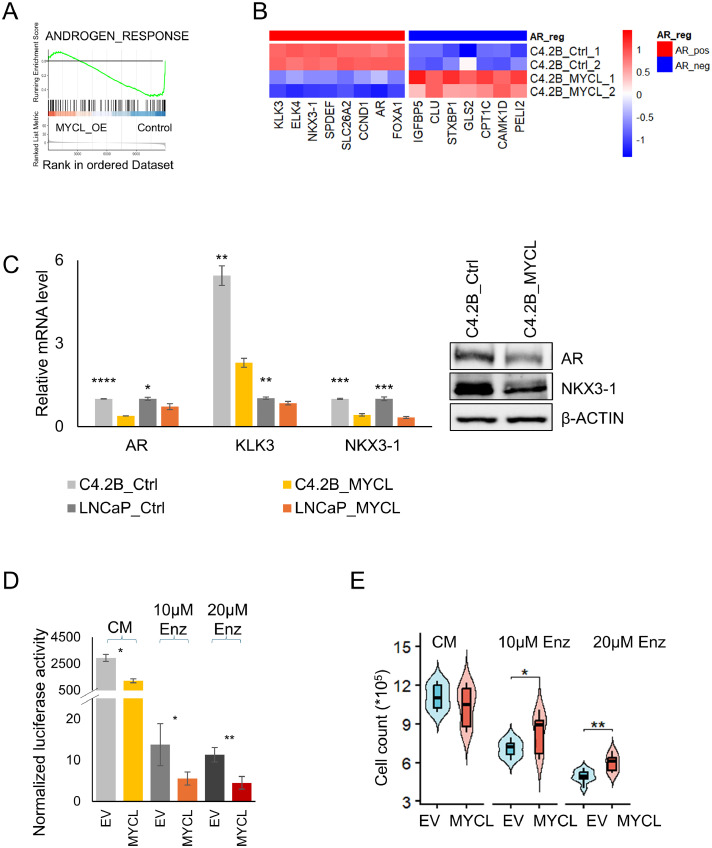
MYCL suppresses androgen receptor signaling and promotes resistance to AR pathway inhibition (A) Gene set enrichment analysis demonstrating significant negative enrichment of the HALLMARK_ANDROGEN_RESPONSE pathway in MYCL-overexpressing C4-2B cells compared with control cells (FDR = 3.45 × 10⁻⁵), indicating suppression of androgen receptor signaling following MYCL expression. (B) AR-regulated transcriptional program. Heatmap showing log₂(TPM + 1) expression of canonical AR target genes in control and MYCL-overexpressing cells. Genes are categorized as positively regulated (AR_pos) or negatively regulated (AR_neg) AR targets. Differentially expressed genes were filtered using thresholds of |log₂FC| ≥ 0.25 and FDR < 0.05. MYCL overexpression results in global attenuation of AR signaling together with upregulation of genes typically induced following androgen deprivation therapy. (C) Molecular validation of AR signaling suppression. RT-qPCR analysis demonstrates reduced expression of AR target genes (AR, KLK3, and NKX3-1) in MYCL-overexpressing cells relative to controls. Immunoblot analysis confirms decreased AR and NKX3-1 protein levels, with β-ACTIN used as a loading control. (D) AR transcriptional activity assay. Luciferase reporter analysis measuring normalized AR transcriptional activity in empty vector (EV) and MYCL-expressing C4-2B cells cultured in control medium (CM) or treated with enzalutamide (ENZ; 10 µM and 20 µM). MYCL expression significantly reduces AR transcriptional activity under basal conditions and further suppresses AR-driven reporter activity following pharmacologic AR inhibition. (E) Cell proliferation under androgen pathway inhibition after 6 days of treatment. Violin-box plots show cell counts of control and MYCL-overexpressing C4-2B cells cultured in CM or treated with increasing concentrations of enzalutamide (10 µM and 20 µM) for 6 days. Although MYCL-expressing cells display reduced basal proliferation, they show relatively less reduction in cell numbers following enzalutamide treatment compared with control cells, consistent with decreased dependence on AR signaling.

Gene set enrichment analysis revealed significant negative enrichment of the Hallmark androgen response pathway in MYCL-overexpressing C4-2B cells compared with controls (Fig. 4A). Consistently, heatmap analysis demonstrated coordinated repression of canonical AR-positively regulated genes, including KLK3, NKX3-1, SPDEF, as well as AR and its binding partner, FOXA1 (Fig. 4B). In parallel, AR-negatively regulated genes such as IGFBP5 and CLU, which are typically upregulated following androgen deprivation, were elevated in MYCL-overexpressing cells. These findings indicate suppression of AR signaling programs upon MYCL overexpression.

*Data are presented as mean ± SEM. Statistical significance was determined using a two-tailed Student’s test (*P < 0.05, **P < 0.01, ***P < 0.001, ****P < 0.0001).

RT-qPCR analysis confirmed reduced expression of AR and canonical AR target genes, including KLK3, as well as luminal epithelial differentiation marker NKX3-1. Corresponding reductions in AR and NKX3-1 protein levels were validated by immunoblot analysis (Fig. 4C).

Functional assessment using an AR-responsive luciferase reporter assay demonstrated significantly reduced AR transcriptional activity in MYCL-overexpressing cells under basal conditions as well as following pharmacologic AR inhibition with enzalutamide (Fig. 4D, Supplementary Fig. 4C). Consistent with diminished reliance on AR signaling, MYCL-overexpressing cells displayed modestly reduced proliferation under control conditions but exhibited significantly improved proliferative capacity relative to control cells at increasing concentrations of enzalutamide (Fig. 4E).

Together, these findings indicate that MYCL overexpression alone is sufficient to suppress AR-driven luminal transcriptional programs and promote a transition toward AR-independent growth states, thereby reducing cellular sensitivity to AR pathway inhibition.

### MYCL is required to maintain neuroendocrine lineage identity in NEPC cells

**Fig. 5:**
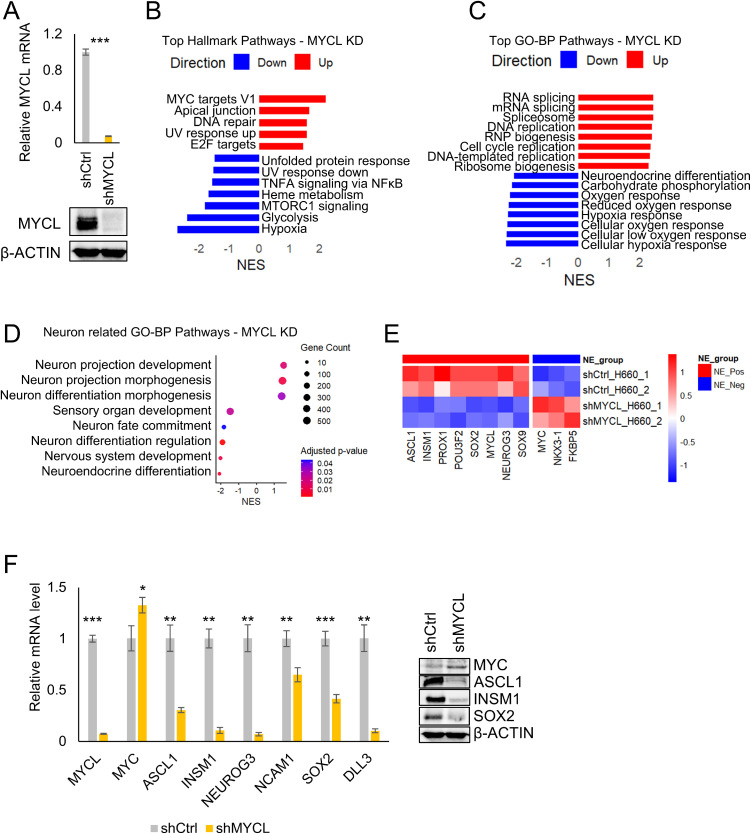
MYCL is required to maintain neuroendocrine lineage identity in NEPC cells (A) Validation of MYCL knockdown in NCI-H660 cells. Relative MYCL mRNA expression measured by RT-qPCR confirms efficient MYCL depletion compared with Control (shCtrl). Immunoblot analysis further verifies reduced MYCL protein expression, with β-ACTIN serving as a loading control. (B) Hallmark pathway enrichment analysis following MYCL knockdown. GSEA of MSigDB Hallmark pathways in MYCL-depleted cells versus controls (FDR < 0.05). Positively enriched pathways include MYC targets, DNA repair, and G2/M checkpoint, whereas negatively enriched pathways include hypoxia, glycolysis, mTORC1 signaling, and inflammatory signaling. (C) Gene Ontology Biological Process enrichment analysis following MYCL depletion (FDR < 0.05). Upregulated pathways are associated with RNA processing, DNA replication, and chromosomal organization, whereas downregulated pathways involve stress adaptation and metabolic processes. (D) Neuron-related GO Biological Process enrichment analysis following MYCL knockdown. Neuron-associated pathways were filtered (FDR < 0.05) and reduced by semantic similarity clustering (similarity cutoff = 0.7). Dot size represents gene count and color indicates adjusted P value. MYCL depletion reduces enrichment of pathways linked to neuron differentiation, nervous system development, and neuroendocrine cell differentiation, with relative enrichment of neuron projection and cellular morphogenesis programs. (E) Heatmap showing differential expression of neuroendocrine lineage markers and adenocarcinoma-associated genes displayed as log₂(TPM + 1). Genes were filtered using thresholds of |log₂FC| ≥ 0.5 and FDR < 0.05. MYCL depletion results in coordinated downregulation of neuroendocrine regulators (ASCL1, INSM1, POU3F2/BRN2, SOX2, NEUROG3) and increased expression of epithelial and AR-associated genes (NKX3-1, FKBP5, and MYC), consistent with loss of neuroendocrine identity and partial reactivation of adenocarcinoma-like transcriptional programs. (F) Molecular validation of lineage reprogramming. RT-qPCR analysis confirms reduced expression of neuroendocrine markers (ASCL1, INSM1, NEUROG3, NCAM1, SOX2, and DLL3) following MYCL knockdown, accompanied by increased MYC expression. Immunoblot analysis validates corresponding protein-level changes.

To directly test whether MYCL sustains NE lineage programs, MYCL was depleted in the NEPC cell line NCI-H660. Efficient knockdown of MYCL was confirmed at both the mRNA and protein levels (Fig. 5A). Global transcriptomic profiling revealed widespread gene-expression reprogramming following MYCL loss. Gene set enrichment analysis of Hallmark pathways demonstrated enrichment of MYC target gene programs and apical junction pathways upon MYCL depletion, accompanied by reduced enrichment of mTORC1 signaling, glycolysis, and hypoxia pathways (Fig. 5B), indicating attenuation of metabolic and stress-adaptive transcriptional programs characteristic of NEPC cells.

*Data are presented as mean ± SEM. Statistical significance was determined using a two-tailed Student’s test (*P < 0.05, **P < 0.01, ***P < 0.001, ****P < 0.0001).

Consistent with this shift, Gene Ontology analysis revealed significant downregulation of biological processes associated with neuronal fate commitment and NE differentiation, together with relative enrichment of DNA replication and cell-cycle associated programs (Fig. 5C), suggesting partial reactivation of proliferative adenocarcinoma-like transcriptional states. Semantic similarity-reduced pathway analysis further demonstrated loss of mature neuronal and NE differentiation signatures, whereas pathways linked to cellular morphogenesis and structural remodeling remained relatively enriched (Fig. 5D), indicating disruption of stabilized NE lineage programs rather than generalized neuronal activation.

Heatmap analysis confirmed coordinated downregulation of canonical NE lineage regulators following MYCL depletion, accompanied by re-expression of epithelial and adenocarcinoma-associated genes, including NKX3-1, FKBP5, and MYC, which are typically suppressed in NEPC (Fig. 5E). These transcriptional changes were validated by RT-qPCR and immunoblot analyses, demonstrating increased expression of MYC and reduced expression of key NEPC regulators ASCL1, INSM1, NEUROG3, NCAM1, SOX2, and DLL3 upon MYCL knockdown (Fig. 5F). Similar results were obtained using an independent MYCL-targeting construct.

Collectively, these findings establish MYCL as a critical lineage-maintenance factor required to preserve NE identity and prevent transcriptional reversion toward an adenocarcinoma-like state in NEPC cells.

### Epigenetic and transcriptional mechanisms regulate MYCL expression in neuroendocrine prostate cancer

To determine whether elevated MYCL expression in NEPC is driven by genomic alterations, copy-number data from the Beltran cohort and PCa cell line datasets from the CCLE were analyzed. No MYCL amplification was detected in NEPC samples, indicating that MYCL overexpression is not associated with copy-number variation and instead likely arises through transcriptional regulation (Supplementary Fig. 5A, B). Similarly, although MYC amplification was observed in NCI-H660 cells, MYC mRNA expression remained relatively low, further suggesting that MYC family gene expression is not solely determined by copy-number status but is influenced by transcriptional regulation (Supplementary Fig. 5B).

**Fig. 6:**
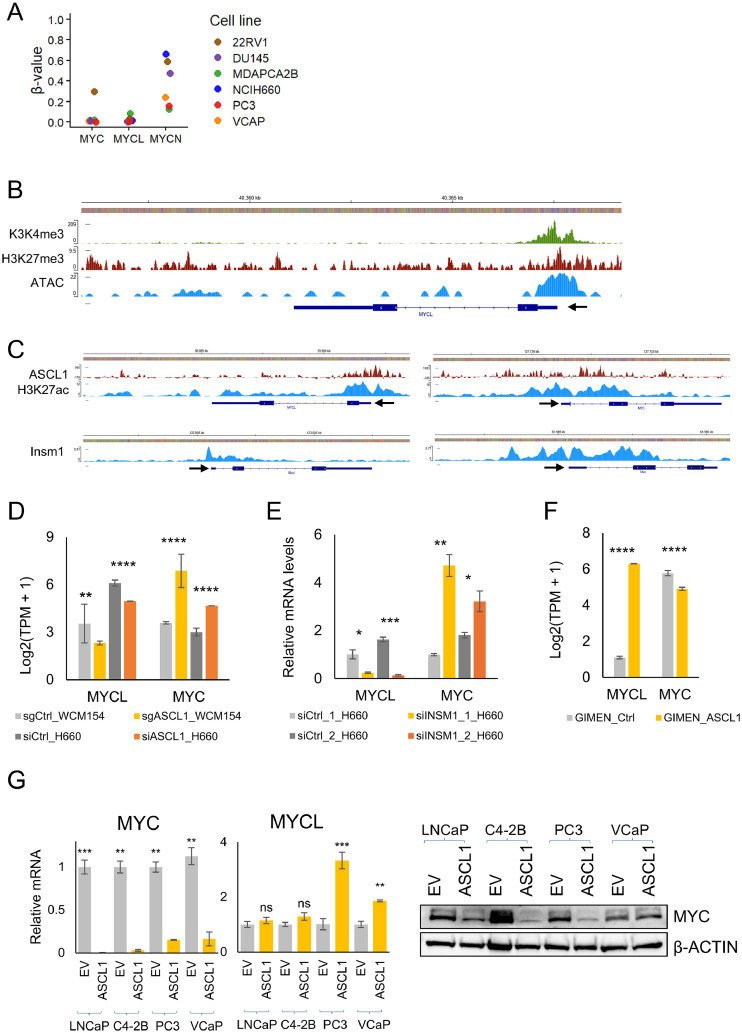
Epigenetic and transcriptional regulation of MYCL within neuroendocrine lineage programs (A) CCLE RRBS analysis of CpG methylation at transcription start site (TSS) clusters across prostate cancer cell lines showing hypomethylation of the MYCL and MYC promoters and hypermethylation of MYCN, indicating differential epigenetic regulation among MYC family genes. β-value plots display DNA methylation levels at MYC, MYCL, and MYCN loci across prostate cancer cell lines (22Rv1, DU145, MDAPCa2B, NCI-H660, PC3, and VCaP). (B) ChIP-seq and ATAC-seq profiles showing chromatin accessibility and histone modifications at the MYCL locus in LNCaP cells (hg38). Co-enrichment of activating H3K4me3 and repressive H3K27me3 marks indicates a bivalent, poised chromatin state, while ATAC-seq signal highlights open chromatin regions permissive for transcriptional activation. (C) Genome browser tracks showing ASCL1 binding at the MYCL and MYC promoter regions together with H3K27ac enrichment in NCI-H660 cells (hg19). INSM1 binding at MYCL and MYC loci in mouse pancreatic β cells (mm10), where both genes are expressed, supports potential transcriptional regulation by neuroendocrine lineage transcription factors. (D) Expression analysis of MYCL and MYC following ASCL1 knockdown in NEPC patient-derived organoids (WCM154) and NCI-H660 cells, showing reduced MYCL expression accompanied by reciprocal changes in MYC. (E) RT-qPCR analysis in NCI-H660 cells demonstrating decreased MYCL and increased MYC expression following INSM1 knockdown, suggesting a reciprocal regulatory relationship between MYCL and MYC. (F) Expression of MYCL and MYC in neuroblastoma-derived GIMEN cells demonstrating induction of MYCL and suppression of MYC following ASCL1 overexpression. (G) RT-qPCR and immunoblot analysis of LNCaP, C4-2B, PC3 and VCaP cells infected with control (EV), ASCL1 adenoviruses showing suppression of MYC following ASCL1 overexpression, suggesting that neuroendocrine lineage transcription factors are associated with repression of MYC in adenocarcinoma cells.

To investigate potential epigenetic regulation, CCLE reduced representation bisulfite sequencing (RRBS) data were analyzed to assess CpG methylation at transcription start site (TSS) clusters across PCa cell lines. The TSS regions of both MYCL and MYC were consistently hypomethylated, suggesting a transcriptionally permissive epigenetic state (Fig. 6A). In contrast, the MYCN promoter displayed predominant hypermethylation, with the exception of 22Rv1 cells, consistent with epigenetic repression. These findings demonstrate differential methylation-based regulation among MYC family members and suggest that the MYCL locus remains broadly accessible for transcription across PCa subtypes.

To further evaluate chromatin accessibility, publicly available ATAC-seq and ChIP-seq datasets from LNCaP cells were examined. The MYCL promoter exhibited open chromatin together with a bivalent chromatin configuration, marked by the co-occurrence of activating H3K4me3 and repressive H3K27me3 histone modifications (Fig. 6B). Such bivalent chromatin states are characteristic of lineage-regulatory genes poised for activation during cellular plasticity, suggesting that MYCL is epigenetically primed for transcriptional activation during NE differentiation.

MYCL expression strongly correlated with the NE transcription factors ASCL1 and INSM1 (Supplementary Figs. 2 and 3). ChIP-seq analysis further revealed ASCL1 binding at the MYCL promoter in NEPC cells (NCI-H660), as well as INSM1 binding at the MYCL locus in mouse pancreatic β cells-an endocrine lineage in which MYCL and INSM1 are co-expressed-supporting conserved transcriptional regulation (Fig. 6C). Notably, both ASCL1 and INSM1 also occupied the MYC locus, suggesting coordinated and reciprocal regulation of MYCL and MYC by NE lineage-defining transcription factors. Functional validation in NEPC patient-derived organoids (WCM154) and NCI-H660 cells demonstrated that knockdown of ASCL1 or INSM1 reduced MYCL expression while concomitantly increasing MYC expression (Fig. 6D, E), indicating opposing regulation of MYC family members within the NE transcriptional network.

To determine whether this transcriptional program extends beyond PCa, RNA-seq datasets from other NE tumor models were analyzed. ASCL1 overexpression in neuroblastoma-derived GI-ME-N cells increased MYCL expression and suppressed MYC levels (Fig. 6F), supporting the existence of a conserved regulatory axis operating in NE tumor contexts. Consistent with this, analysis of lung cancer cell lines showed that MYCL expression was significantly elevated in small-cell lung cancer (SCLC) compared with non-small cell lung cancer (NSCLC), in line with its association with NE lineage identity (Supplementary Fig. 6A). MYCL expression also positively correlated with NE markers and inversely correlated with MYC expression (Supplementary Fig. 6B, C). Moreover, RNA-seq analysis following ASCL1 knockdown in SCLC cell lines (NCI-H2107, NCI-H209, and DMS53) demonstrated significant downregulation of MYCL expression (Supplementary Fig. 6D).

Overexpression of ASCL1 suppressed MYC in all AdPC models tested but did not consistently induce MYCL expression. It failed to upregulate MYCL in LNCaP or C4-2B cells (Fig. 6G; Supplementary Fig. 7). In contrast, MYCL mRNA induction was observed in the more aggressive PC3 and VCaP models, suggesting that additional regulatory mechanisms are required for MYCL activation.

Collectively, these findings demonstrate that elevated MYCL expression in NEPC is not driven by genomic amplification but instead arises from a permissive epigenetic landscape and is linked to neuroendocrine lineage transcriptional programs associated with ASCL1 and INSM1. The reciprocal regulation of MYCL and MYC suggests the existence of a MYC family regulatory switch that promotes and stabilizes neuroendocrine lineage identity across multiple cancer contexts.

## Discussion

NEPC represents a highly aggressive and therapy-resistant subtype of PCa that most commonly arises through lineage plasticity from prostate adenocarcinoma under selective pressure imposed by AR pathway inhibitors [3,13]. Unlike classical tumor progression driven primarily by the accumulation of new oncogenic mutations, this transdifferentiation process is largely governed by epigenetic remodeling and reprogramming of transcriptional networks [14,15]. A defining hallmark of NEPC is the loss of AR signaling accompanied by activation of NE lineage programs orchestrated by master transcription factors such as ASCL1 and INSM1 [16,17]. However, the transcriptional effectors that contribute to the establishment and maintenance of the NE state remain incompletely understood.

Here, we identify MYCL as a previously underappreciated, lineage-restricted member of the MYC family that is selectively upregulated in NEPC and contributes to the establishment and maintenance of NE lineage identity. In contrast to MYC, which is commonly elevated in AdPC and closely associated with AR-dependent proliferative signaling [[18], [19], [20]]. MYCL expression is markedly enriched in NEPC and shows strong concordance with canonical NE markers across multiple patient cohorts, patient-derived models, and experimental systems. This divergent expression pattern suggests a functional reassignment of MYC family dependency during lineage plasticity.

Our findings support a model in which MYCL functions as an effector of the NE lineage program operating within a permissive epigenetic environment and contributing to suppression of AR signaling. The MYCL locus exhibits features of transcriptional accessibility and a poised chromatin configuration that may facilitate activation in NE contexts by lineage transcription factors, accompanied by reduced MYC expression. Importantly, MYCL regulation occurs independently of genomic amplification, indicating that transcriptional and epigenetic mechanisms-rather than genetic alterations-primarily drive its activation in NEPC. These observations position MYCL not merely as a marker of NE differentiation but as a lineage-stabilizing transcriptional amplifier that reinforces NEPC gene expression programs while opposing AR-driven transcriptional states. Collectively, these findings support a model of a MYC family regulatory switch during lineage plasticity, in which MYCL replaces MYC to sustain NE lineage identity.

### A lineage-associated MYC family switch in prostate cancer progression

MYC family oncogenes have long been implicated in PCa pathogenesis [18]; however, their lineage-specific deployment during disease progression has remained poorly defined. MYC amplification and overexpression are hallmarks of AdPC, where MYC drives proliferation, metabolic reprogramming, and AR signaling output [6,7]. In contrast, MYCN amplification has been reported in a subset of NEPC cases [8], where it cooperates with AKT signaling and epigenetic regulators such as EZH2 to suppress AR activity and promote NE features [9,10].

Our data reveal a distinct and complementary paradigm in which MYCL is selectively and robustly upregulated in NEPC, while MYC is concurrently suppressed and MYCN remains low at the transcript level in patient samples and at both transcript and protein levels in the experimental models examined. This reciprocal MYCL-MYC expression pattern is consistently observed across multiple patient cohorts, bulk and single-cell transcriptomic datasets, patient-derived xenografts, and PCa cell line models. Notably, MYCL expression is detectable not only in NEPC cells but also in AR-high adenocarcinoma subpopulations, suggesting that MYCL activation may precede or accompany early lineage transitions rather than representing a late consequence of fully established NE differentiation. These findings support a model in which PCa progression involves a dynamic reconfiguration of MYC family usage that closely tracks lineage identity and transcriptional state.

Functionally, this MYC family switch reflects divergent transcriptional programs associated with epithelial-luminal versus NE lineages. MYC is highly expressed in AdPC and castration-resistant PCa but is strongly repressed in NEPC. In contrast, MYCL is markedly upregulated in NEPC and correlates inversely with MYC expression and luminal epithelial genes such as NKX3-1 and KLK3, while showing strong concordance with canonical NEPC markers. Importantly, a similar MYCL-associated transcriptional axis has been described in small-cell lung cancer, where MYCL expression aligns with ASCL1-driven NE subtypes [21,22], suggesting that MYCL-associated programs may represent a conserved regulatory mechanism across NE malignancies.

### MYCL as a driver of cytoskeletal remodeling and neuroendocrine plasticity

Unlike MYC, whose oncogenic activity is tightly coupled to cell-cycle progression and proliferative output [5], MYCL overexpression in PCa cells exerted minimal effects on overall growth rates. Instead, MYCL markedly reduced cell adhesion and induced transcriptional changes in genes associated with cytoskeletal regulation. Coordinated downregulation of integrin components, together with altered expression of cell-cycle regulators, was accompanied by changes in components of the Rho-Rac GTPase signaling, Ephrin-EPH pathways, and mesenchymal markers, suggesting a role for MYCL in regulating cell-cell and cell-matrix interactions and cellular architecture. Consistently, MYCL knockdown in NEPC models also altered pathways associated with morphogenesis and cytoskeletal organization. These phenotypic and transcriptional changes are notable given emerging evidence that disruption of extracellular matrix-integrin signaling and cell adhesion can promote NE lineage plasticity in PCa [23]. In NEPC, cells characteristically adopt rounded morphologies and exhibit reduced substrate attachment. Our data suggest that MYCL contributes to these traits by reprogramming adhesion and cytoskeleton-related gene networks, thereby promoting lineage flexibility and facilitating adaptation to androgen receptor-depleted environments.

### MYCL suppresses AR signaling and MYC-dependent transcriptional programs

A defining feature of NEPC is the loss of AR signaling and luminal epithelial identity [8]. We demonstrate that MYCL overexpression is sufficient to suppress AR transcriptional output, downregulate canonical AR target genes, and attenuate AR-driven reporter activity. Consistent with this transcriptional rewiring, MYCL-expressing cells exhibit increased tolerance to AR pathway inhibition, supporting a functional shift toward AR-independent survival strategies.

Concomitant with AR suppression, MYCL induces broad repression of MYC-dependent transcriptional programs and reduces MYC expression at both the mRNA and protein levels. This finding reveals a functional antagonism between MYCL and MYC that extends beyond correlative lineage association. Rather than acting redundantly, MYCL appears to actively displace MYC-driven programs linked to proliferation, cell-cycle progression, and genome maintenance, while favoring differentiation-associated, neuronal, and stress-adaptive transcriptional states. This functional divergence provides a mechanistic basis for the MYC family switch observed during NE transdifferentiation.

Notably, a similar division of labor among MYC family members has been described in small-cell lung cancer, where MYCL promotes neuronal-like transcriptional programs but is insufficient on its own to fully establish ASCL1-positive NE lineage identity [21]. In that context, replacement of MYC with MYCL shifts the transcriptional landscape toward NE gene expression, particularly when MYC activity is diminished, and is accompanied by reconfiguration of chromatin accessibility at neuronal regulatory elements [21]. Together, these observations highlight a context-dependent role for MYCL, distinct from canonical MYC, in suppressing proliferative, lineage-incompatible programs while reinforcing NE identity.

### MYCL is required to maintain neuroendocrine lineage identity

Loss-of-function studies in the NEPC cell line NCI-H660 demonstrate that MYCL is not only sufficient to induce NE-like programs but is also required to maintain them. MYCL depletion resulted in coordinated downregulation of canonical NEPC markers, including ASCL1, INSM1, POU3F2, NEUROG3, SOX2, and DLL3, alongside reactivation of epithelial and adenocarcinoma-associated genes such as NKX3-1, FKBP5, and MYC. This transcriptional reversion was accompanied by reactivation of DNA replication and proliferation-associated programs, suggesting that MYCL actively enforces a differentiated, AR-independent NE state.

Notably, MYCL knockdown did not simply abolish neuronal gene expression but instead disrupted mature NE lineage programs while disturbing morphogenesis-related pathways. This distinction highlights MYCL’s role in stabilizing lineage identity, consistent with its function as a lineage maintenance factor.

### Epigenetic regulation of MYCL

A key insight from this study is that activation of MYCL in NEPC is not driven by genomic amplification. In contrast to MYC, which is frequently upregulated through copy-number gain in AdPC [7], the MYCL locus resides within an epigenetically permissive chromatin landscape characterized by promoter hypomethylation and bivalent chromatin features. Such chromatin configurations are commonly observed at lineage-specifying genes in developmental systems, where they enable rapid transcriptional activation during cellular differentiation and lineage reprogramming [24]. This epigenetic priming may facilitate inducible MYCL activation during transition toward the NE state.

Our data further implicate ASCL1 and INSM1, core NE lineage transcription factors, as regulators associated with MYCL expression. ChIP-seq analyses demonstrate binding of both ASCL1 and INSM1 at the MYCL locus, while functional perturbation experiments across prostate and lung NE cancer models show that depletion of either factor reduces MYCL expression and is accompanied by reciprocal upregulation of MYC. Conversely, perturbation of MYCL also alters ASCL1 and INSM1 expression levels, suggesting the presence of a reinforcing regulatory relationship rather than a strictly linear transcriptional hierarchy. Although additional mechanistic studies will be required to determine whether ASCL1 and INSM1 function as direct upstream regulators or components of a feedback network, these findings support the existence of an ASCL1-INSM1-associated transcriptional network linked to MYCL expression.

Within this framework, MYCL likely functions downstream of NE transcriptional programs to reinforce lineage-specific gene expression while antagonizing MYC-dependent adenocarcinoma-associated transcriptional states. Such reciprocal regulation provides a mechanistic basis for the MYC family switch observed during NE transdifferentiation, whereby MYCL replaces MYC as the dominant MYC paralog supporting lineage identity.

Importantly, this regulatory architecture appears conserved across NE malignancies. In small-cell lung cancer, MYCL expression is selectively enriched relative to non-small cell lung cancer and strongly associates with ASCL1-positive NE subtypes. Previous studies have demonstrated mutually exclusive amplification of MYC family members in SCLC and shown that MYC, MYCN, and MYCL drive distinct lineage-associated transcriptional programs [21]. In this context, MYCL preferentially supports neuronal and NE transcriptional programs, whereas MYC is linked to proliferative and metabolic gene networks.

Together, these findings support a model in which MYC-to-MYCL switching represents a conserved mechanism of NE reprogramming, enabling stabilization of lineage commitment across tumor types. The reciprocal regulatory interactions observed between MYCL and NE transcription factors further suggest the presence of a positive feedback circuit that reinforces and maintains NE identity once established.

### Biological and therapeutic implications

Although MYCL amplification is not a prominent feature in the Beltran NEPC cohort, prior studies have reported focal MYCL amplification in clinically localized prostate tumors [11]. These events were more frequent than MYC amplification and mutually exclusive with other MYC family amplifications. Importantly, MYCL expression remained low in these tumors, suggesting epigenetic silencing in AdPC, which is unlocked during lineage reprogramming.

Interestingly, MYCL has also been shown to promote iPSC reprogramming more efficiently than MYC and MYCN, without inducing tumor formation [25]. This separation of reprogramming capacity from oncogenic transformation supports the idea that MYCL functions as a lineage-specifying factor rather than a classical oncogene. In NEPC, its upregulation in NEPC may reflect its association with transcriptional rather than proliferative signatures.

Given the longstanding challenges associated with directly targeting MYC family proteins therapeutically, modulation of regulatory pathways associated with MYCL expression may represent a more feasible strategy. Transcription factors linked to NE lineage programs, including ASCL1 and INSM1, together with epigenetic mechanisms that maintain a permissive chromatin landscape, may therefore represent potential therapeutic entry points influencing MYCL expression. Consistent with this concept, MYCL silencing in small-cell lung cancer models has been shown to suppress tumor development [26], supporting the functional importance of MYCL and highlighting its potential vulnerability across NE malignancies.

## Conclusions

This study identifies MYCL as a lineage-associated regulator that drives and maintains NE lineage identity in PCa. In contrast to MYC, which predominates in adenocarcinoma and supports AR-dependent proliferative programs, MYCL is selectively upregulated in NEPC and coincides with suppression of both AR signaling and MYC-dependent transcriptional networks. Functional perturbation experiments demonstrate that MYCL promotes NE-like transcriptional reprogramming, remodels cytoskeletal and adhesion programs associated with cellular plasticity, and reduces dependence on AR signaling, whereas MYCL depletion disrupts NE lineage programs and restores adenocarcinoma-associated gene expression, including MYC. Mechanistically, MYCL activation and MYC suppression arise from a permissive epigenetic landscape together with transcriptional regulation by NE lineage factors such as ASCL1 and INSM1. Together, these findings define a MYC family regulatory switch in which MYCL replaces MYC to stabilize NE lineage programs during PCa progression.

## Methods

### Bioinformatic analysis

To investigate the expression of MYC family genes and NE markers across different PCa states, RNA-seq data from patient cohorts including Beltran and SU2C were obtained from cBioPortal [27,28]. The Labrecque mCRPC and LuCaP datasets were obtained from GSE126078 [29]. RNA-seq data from metastatic castration-resistant PCa cohorts generated by the West Coast Dream Team (WCDT) collaboration were obtained from the UCSF Quigley Laboratory data portal and analyzed as previously described [30] and the ProAtlas dataset was previously published by our laboratory [31]. Gene expression data from PCa cell lines were obtained from our laboratory-published CTPC dataset [32] and were included for comparative analysis of MYC family genes and NE markers. Single-cell RNA-seq plots were obtained from the Human Prostate Single Cell Atlas (HuPSA), which was generated using scRNA-seq data from multiple studies, including publicly available cohorts and datasets produced by our research team [31].

Violin-box plots were generated to visualize expression patterns of MYC family members across disease subtypes. Statistical differences between groups were assessed using a two-tailed Wilcoxon rank-sum test (*P < 0.05, **P < 0.01, ***P < 0.001, ****P < 0.0001). Heatmaps were generated to visualize expression patterns of NE markers, AdPC-enriched genes, and MYC family members. Scatter plots were used to assess correlations between MYC, MYCL, and NE marker expression across pathological subtypes. Normalized gene expression values were expressed as log₂(TPM + 1). Data visualization was performed using the ggplot2 package, and heatmaps were generated using the pheatmap R package.

For the Beltran cohort and cancer cell lines, copy-number variation and gene expression data for MYC family genes were obtained from cBioPortal. CCLE RRBS-derived promoter methylation β-values at transcription start site (TSS) clusters for MYC family genes were obtained from the CCLE dataset. Data were visualized in R using ggplot2 as jittered dot plots, with points colored by cell line. MYCL expression in NSCLC and SCLC cell lines was visualized using boxplots based on log₂(TPM + 1) values from CCLE datasets.

Epigenomic datasets were retrieved from GEO, including H3K27me3 and H3K4me3 ChIP-seq profiles in LNCaP cells from GSE148935 [33] and ATAC-seq data in LNCaP cells from GSE139099 [34], INSM1 ChIP-seq data in pancreatic β-cells were obtained from GSE54046 [35] and ASCL1 ChIP-seq data in H660 cells were retrieved from GSE183198 [16]. H3K27ac ChIP-seq data in H660 cells were downloaded from GSE224421 [16]. All downloaded BigWig files were visualized using the Integrative Genomics Viewer (IGV) [36]. Except for INSM1 (mm10) and H660 ASCL1 and H3K27ac datasets (hg38), all data were aligned to the hg19 genome build.

### RNA-seq analysis

Raw gene-level read counts were converted to TPM values by normalizing counts to gene length using Ensembl transcript annotations retrieved via biomaRt, followed by scaling to 1 × 10⁶. Gene expression values were visualized as log₂(TPM + 1) using the pheatmap R package. Differential expression analysis between control and MYCL knockdown or overexpression conditions was performed independently for each cell line using DESeq2 following low-count filtering. Statistical significance was determined using Benjamini–Hochberg adjusted *P* values (padj).

Hallmark and Gene Ontology Biological Process enrichment analyses were performed using the clusterProfiler R package. Gene set enrichment analysis (GSEA) was conducted using ranked DESeq2 test statistics, and pathways with a false discovery rate (FDR) < 0.05 were considered significantly enriched. Neuron-related GO Biological Process terms were identified through keyword-based filtering of significant pathways, and redundancy among neuron-associated terms was reduced using semantic similarity clustering implemented in GOSemSim with a similarity cutoff of 0.7 prior to visualization.

RNA-seq datasets for ASCL1 knockdown in the patient-derived organoid WCM154 and in NCI-H660, H2107, H209, and DMS53 cell lines were obtained from GEO GSE234819 [37], GSE183199 [16], GSE151000 [38], GSE129340 [39], GSE179071 [40]. RNA-seq data for ASCL1 overexpression in GI-ME-N neuroblastoma cells were obtained from GSE214796 [41].

RNA-seq data generated in this study, including MYCL overexpression in C4-2B cells and MYCL knockdown in NCI-H660 cells, are available in the GEO database under accession number GSE324984.

### Cell culture

PCa cells were cultured in RPMI-1640 medium supplemented with 10% fetal bovine serum (FBS) and 1% penicillin-streptomycin at 37 °C in a humidified incubator with 5% CO₂. Cells were passaged upon reaching ∼80% confluency using 0.25% trypsin-EDTA and reseeded at 50% confluency for further experiments. HEK293 cells were cultured in Dulbecco’s Modified Eagle Medium (DMEM) supplemented with 10% FBS and 1% penicillin-streptomycin under the same incubation conditions.

### Plasmid constructs

The MYCL overexpression construct was generated using a lentiviral expression vector encoding human MYCL (NM_001033082.3) under the EF1A promoter (pLV[Exp]-Puro-EF1A>hMYCL/T2A/EGFP; VectorBuilder), which co-expresses EGFP via a T2A sequence. For MYCL knockdown, two independent RNA interference platforms comprising a total of four MYCL-targeting shRNA sequences were used to minimize sequence-specific off-target effects. An adenoviral triple miR30-based shRNA vector (pAV[3miR30]-CMV>NLS-TagBFP2; VectorBuilder) expressing three independent miR30-adapted shRNAs targeting MYCL was used for transient knockdown. A lentiviral shRNA construct targeting human MYCL (pLV[shRNA]-Puro-U6>hMYCL[shRNA#1]; VectorBuilder) was used to generate stable MYCL knockdown cells. INSM1 knockdown was performed using an adenoviral shRNA construct, and adenoviral vectors for INSM1 and ASCL1 overexpression were described previously [42,43].

### Lentiviral overexpression of MYCL

MYCL expression constructs were co-transfected with the ViraPower™ Lentiviral Packaging Mix into 293FT cells using PolyJet™ In Vitro DNA Transfection Reagent (SignaGen Laboratories) according to the manufacturer’s instructions. Lentiviral supernatants were collected and used to infect PCa cells. Transduced cells were selected with puromycin or enriched by fluorescence-based cell sorting three days post-infection.

### Adenoviral infection and gene perturbation

Recombinant adenovirus was generated by transfecting HEK293A cells at approximately 80% confluency with plasmid DNA using PolyJet™ In Vitro DNA Transfection Reagent, according to the manufacturer’s instructions. Upon the appearance of cytopathic effects, viral particles were harvested by three freeze-thaw cycles and amplified in HEK293A cells. After viral titer determination, target cells were infected at a multiplicity of infection (MOI) of 1 for 2 h. Knockdown or overexpression efficiency was assessed 72 h post-infection by RT-qPCR. Adenoviral knockdown of INSM1 and MYCL was carried out in NCI-H660 cells, while adenoviral overexpression of ASCL1 and INSM1 was performed in LNCap, C4-2B, PC3, and VCaP cells.

### RT-qPCR and Western blot analysis

Total RNA was isolated from cells using the Zymo RNA Isolation Kit according to the manufacturer’s instructions. Complementary DNA (cDNA) was synthesized using ABScript III Reverse Transcriptase (Abclonal). RT-qPCR was performed using SYBR Green chemistry with gene-specific primers following the two-step qPCR protocol recommended by the manufacturer. Cycle threshold (Ct) values were analyzed using the ΔΔCt method to determine relative gene expression levels. Statistical analysis of RT-qPCR data was performed using an unpaired two-tailed Student’s t-test applied to fold-change values to compare control and experimental groups. Statistical significance was defined as follows: *P < 0.05, **P < 0.01, ***P < 0.001, ****P < 0.0001.

For Western blot analysis, cells were lysed in Laemmli SDS sample buffer and subjected to SDS-PAGE followed by immunoblotting using standard procedures. Primary antibodies used were β-actin (sc-47778; Santa Cruz Biotechnology), INSM1 (sc-271408; Santa Cruz Biotechnology), MYCL (#76266; Cell Signaling Technology), MYCN (#51705S; Cell Signaling Technology), MYC (#9402S; Cell Signaling Technology), SOX2 (#3728S; Cell Signaling Technology), ASCL1 (556604; BD Pharmingen), AR (#5153S; Cell Signaling Technology), and NKX3-1 (0314; Athena Enzyme Systems). All primary antibodies were used at a dilution of 1:1000. HRP-conjugated secondary antibodies were used for detection. Protein bands were visualized using Pierce™ ECL Western Blotting Substrate (Thermo Scientific) and imaged using the ChemiDoc™ Touch Imaging System (Bio-Rad).

### Cell proliferation and migration assays

Cell proliferation was assessed by manual cell counting and IncuCyte live-cell imaging. Control and MYCL-overexpressing cells were seeded at equal densities in four replicate wells and collected at days 2, 4, 5, and 6. Total and viable cells were quantified using trypan blue exclusion with an automated cell counter. Statistical significance was determined using an unpaired two-tailed Student’s *t*-test.

For live-cell proliferation analysis, cells were seeded in 96-well plates and monitored using the IncuCyte® Live-Cell Analysis System (Sartorius). Cell proliferation was quantified as percent confluence over time using IncuCyte analysis software according to the manufacturer’s instructions.

Cell migration was evaluated using a wound-healing assay performed with the IncuCyte® system following the manufacturer’s (Sartorius) protocol. Wound closure was monitored by live-cell imaging, and migration was quantified as relative wound confluence over time.

### Flow cytometric analysis of cell cycle and apoptosis

Cells were harvested, washed with PBS, and analyzed by flow cytometry for cell-cycle distribution and apoptosis. For cell-cycle analysis, cells were fixed in 70% ethanol, treated with RNase A, and stained with propidium iodide (PI) to measure DNA content. For apoptosis analysis, cells were stained using the APC Annexin V Apoptosis Detection Kit with 7-AAD (BioLegend) according to the manufacturer’s instructions. Briefly, cells were incubated with Annexin V–APC and 7-AAD in Annexin V binding buffer with appropriate compensation controls. Data were acquired on a NovoCyte Quanteon flow cytometer (Agilent/ACEA Biosciences) and analyzed using NovoExpress software, and cell-cycle distribution and apoptotic populations were quantified.

### Cell adhesion assay

For cell adhesion assays, cells were seeded in 12-well plates with six replicates per condition at each time point (18, 24, 48, and 72 h). At the indicated time points following seeding, representative images were acquired, after which non-adherent cells were removed by washing with PBS. Adherent cells were then fixed and stained with crystal violet. After 30 min of incubation, excess stain was removed by washing with water, and plates were allowed to air-dry. Crystal violet was subsequently solubilized by adding 10% acetic acid to each well, and plates were placed on a rocker for at least 24 h. Absorbance was measured at 595 nm to quantify cell adhesion.

### Luciferase reporter assay

Cells were co-transfected with an AR promoter–driven luciferase reporter construct and either a MYCL expression plasmid or control empty vector using PolyJet™ In Vitro DNA Transfection Reagent (SignaGen Laboratories) according to the manufacturer’s instructions. Forty-eight hours post-transfection, cells were lysed and luciferase activity was measured using the Promega Luciferase Assay System. Luciferase signals were normalized to total protein concentration from the same lysates to account for differences in cell number. Experiments were performed in biological triplicates (n = 3).

## Declaration of generative AI and AI-assisted technologies in the writing process

During the preparation of this work the authors used ChatGPT to improve readability. After using this tool, the authors reviewed and edited the content as needed and take full responsibility for the content of the publication.

## CRediT authorship contribution statement

**Jeyaluxmy Sivalingam:** Writing – review & editing, Writing – original draft, Visualization, Validation, Software, Methodology, Investigation, Formal analysis, Data curation, Conceptualization. **Kaleigh Ballagh:** Investigation. **Kyung Hyun Cho:** Data curation. **Yingli Shi:** Data curation. **Lin Li:** Investigation. **Preston Barron:** Data curation. **Michael S. Lan:** Writing – review & editing, Investigation. **Omar E Franco:** Writing – review & editing, Conceptualization. **Xiuping Yu:** Writing – review & editing, Validation, Supervision, Resources, Project administration, Methodology, Investigation, Funding acquisition, Formal analysis, Data curation, Conceptualization.

## Declaration of competing interest

The authors declare no conflict of interest.

## Appendix Supplementary materials

Image, image 1 dummy alt text

Image, image 2 dummy alt text

Image, image 3 dummy alt text

Image, image 4 dummy alt text

Image, image 5 dummy alt text

Image, image 6 dummy alt text

Image, image 7 dummy alt text

Image, application 8

Image, application 9
