# Supplementary figures and images for: A MYC family switch: L-MYC drives and maintains neuroendocrine lineage programs in prostate cancer
Source: Neoplasia. 2026 Apr 17;77:101307. doi: 10.1016/j.neo.2026.101307 (PMC13098336; doi:10.1016/j.neo.2026.101307)

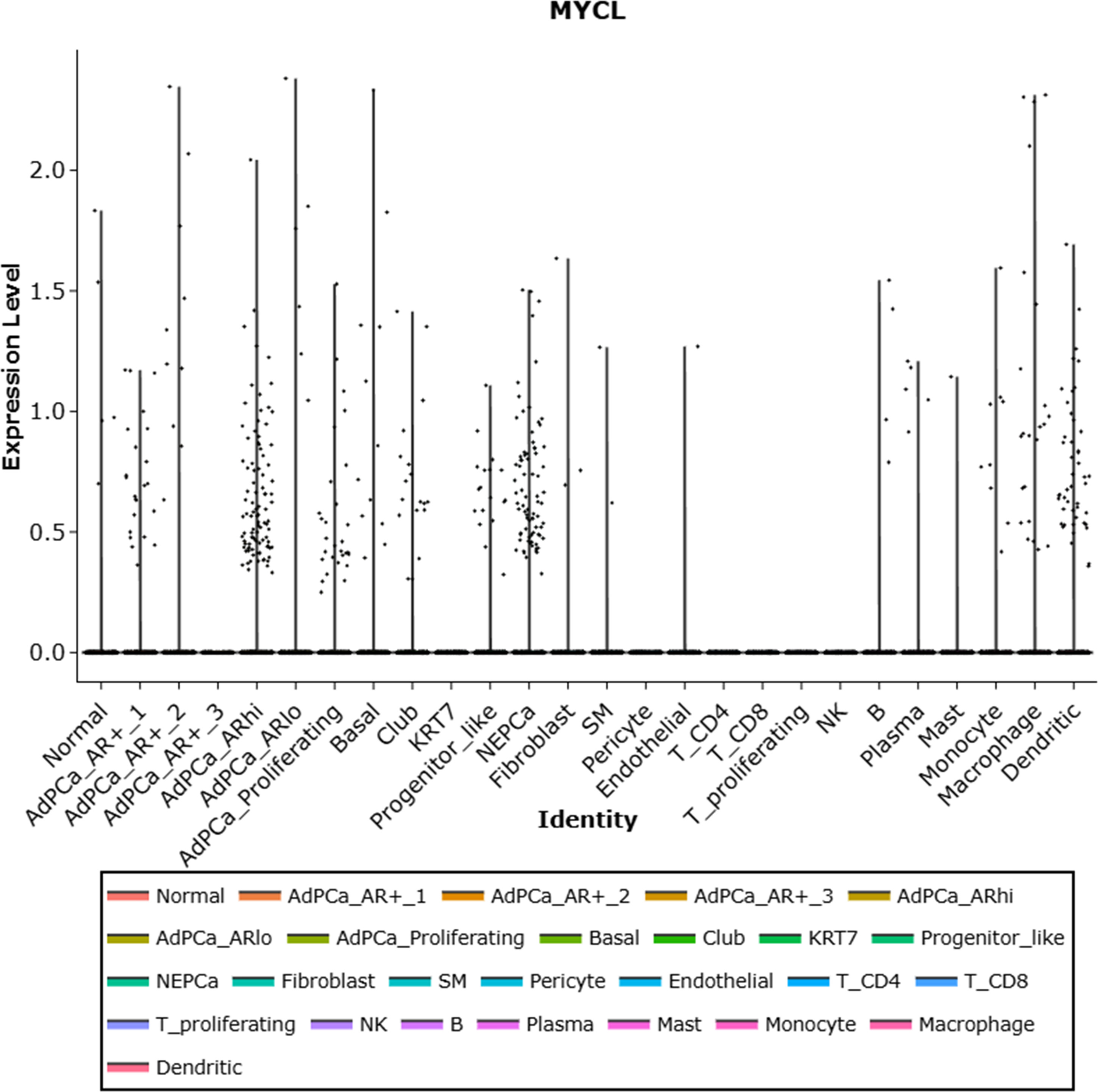

Supplement: Supplementary file 1 [file mmc1.jpg]

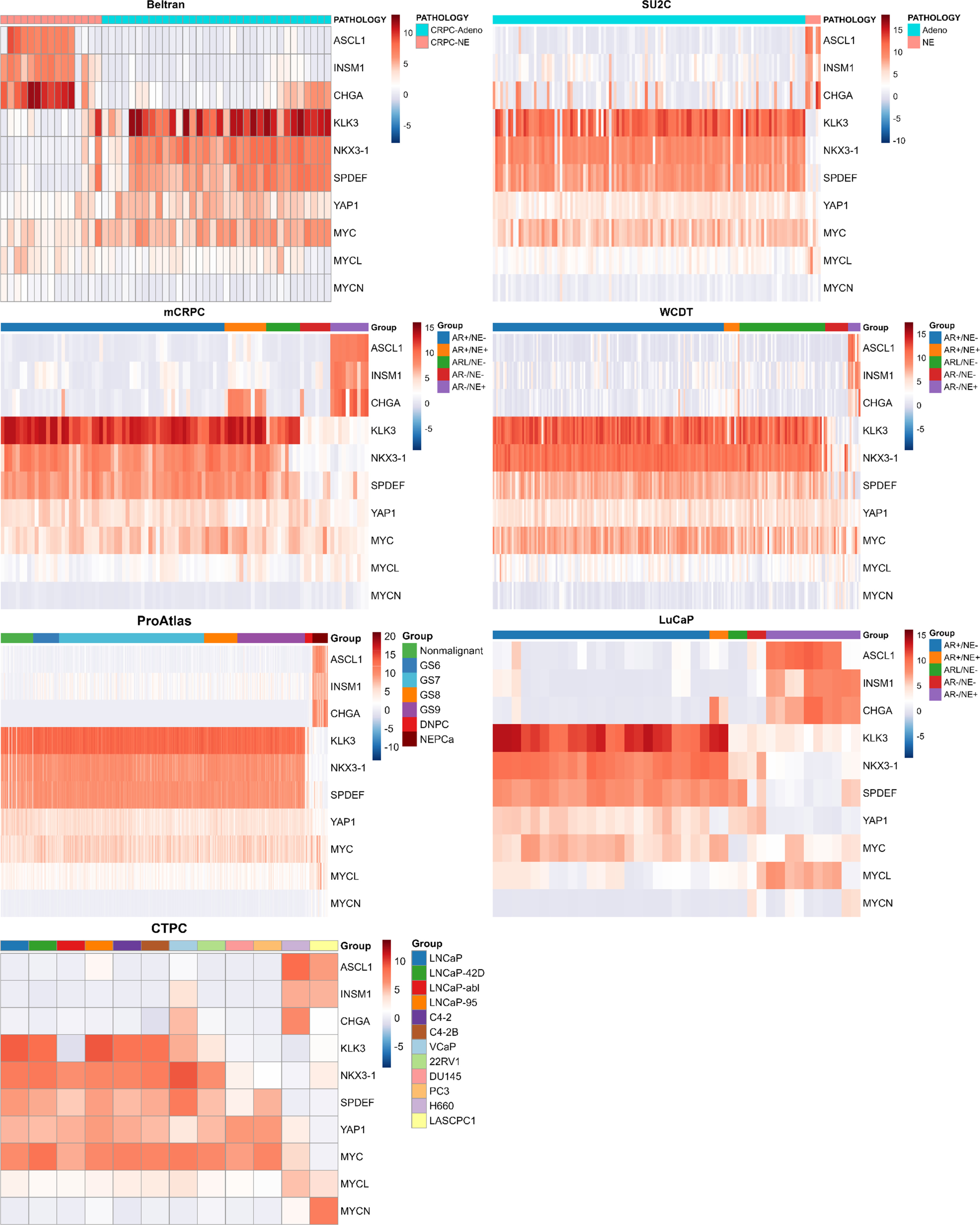

Supplement: Supplementary file 2 [file mmc2.jpg]

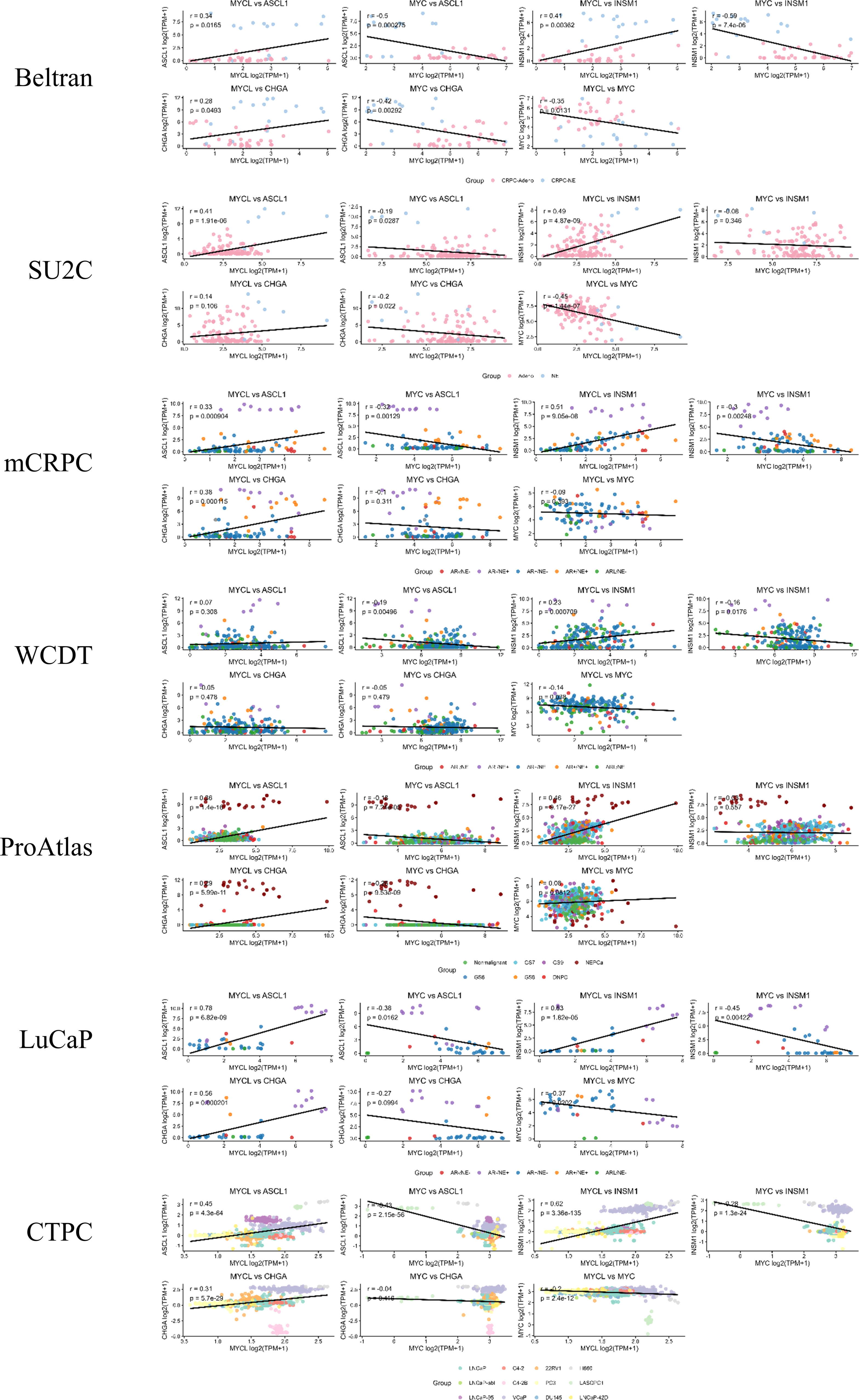

Supplement: Supplementary file 3 [file mmc3.jpg]

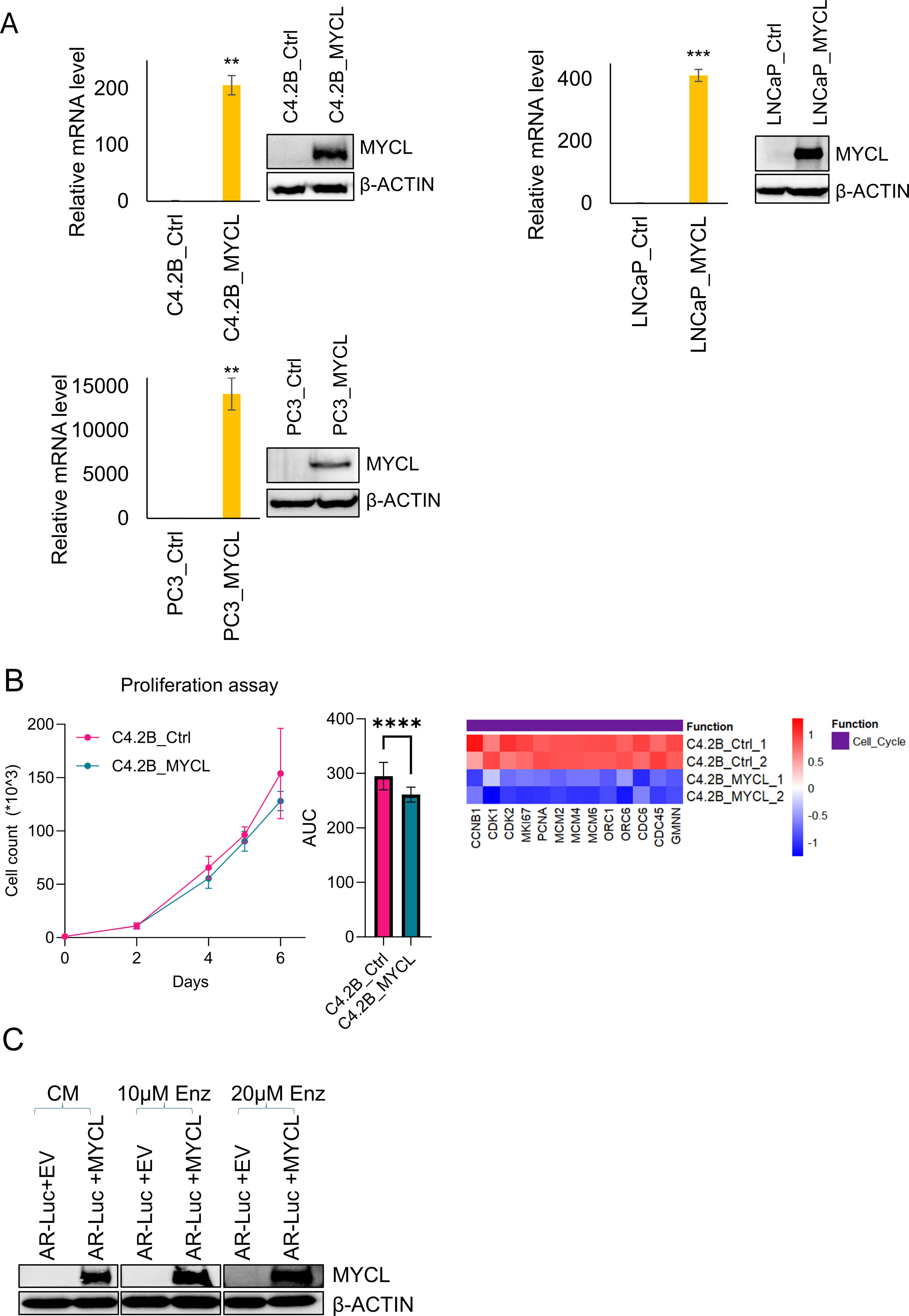

Supplement: Supplementary file 4 [file mmc4.jpg]

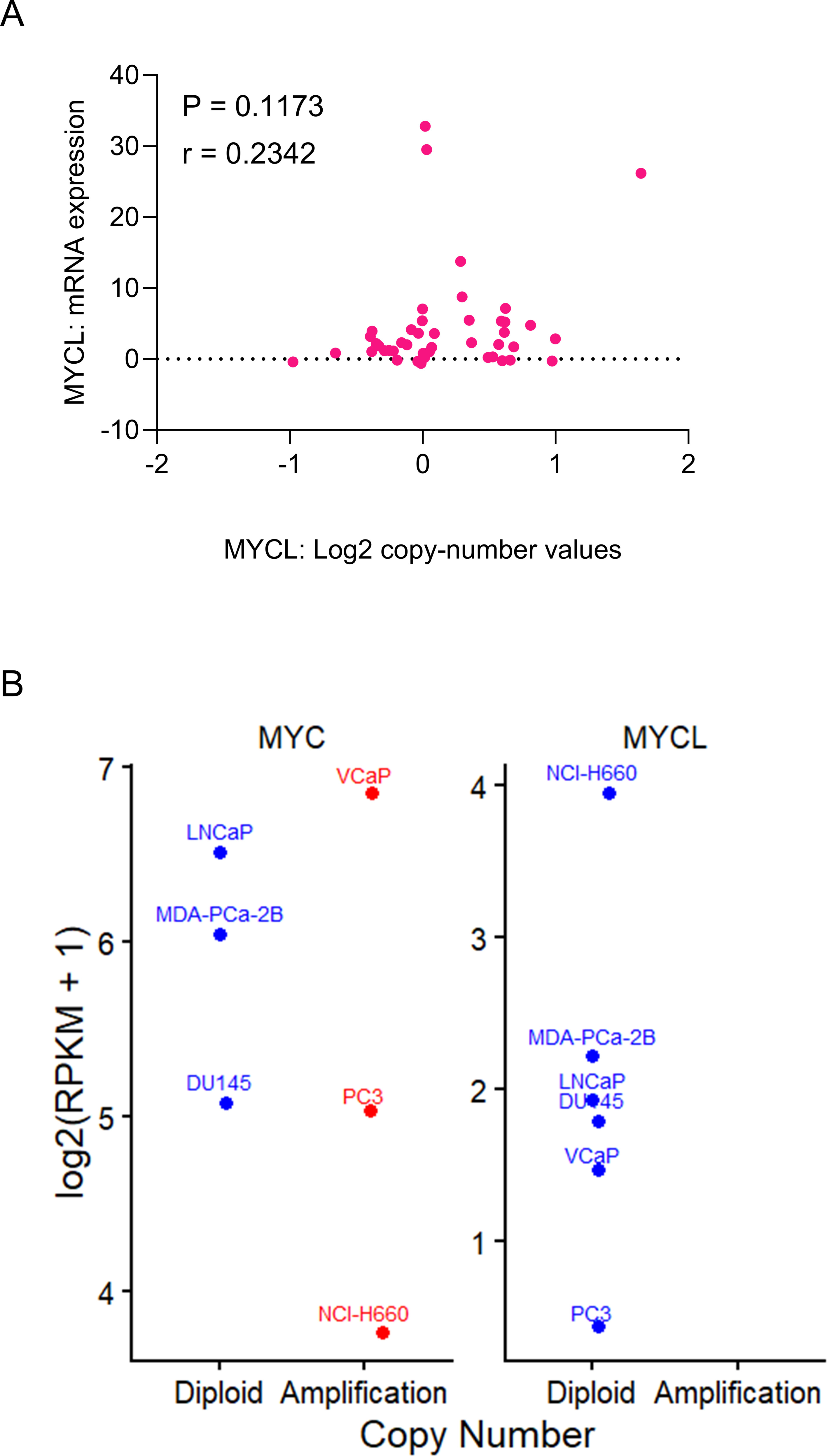

Supplement: Supplementary file 5 [file mmc5.jpg]

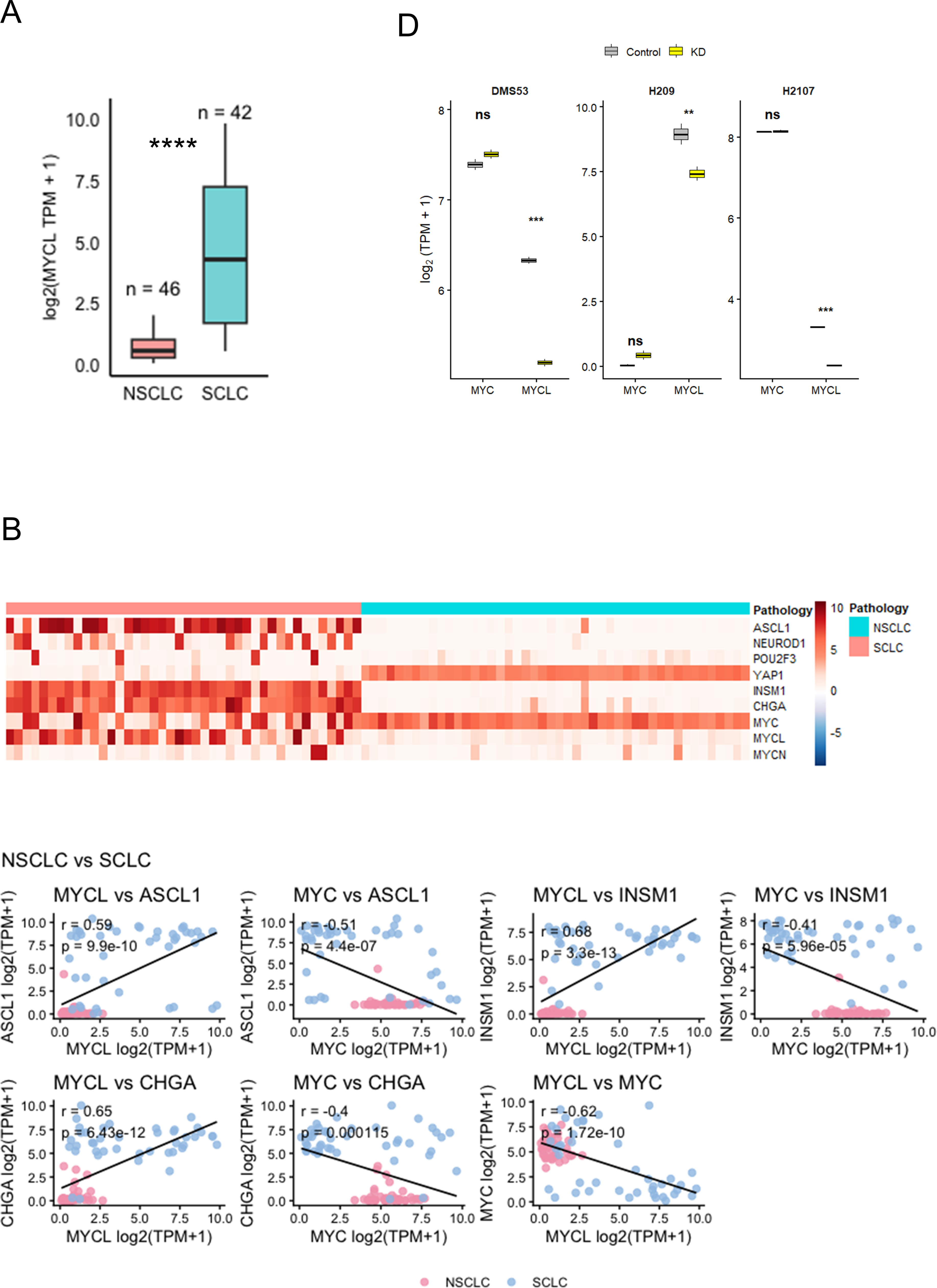

Supplement: Supplementary file 6 [file mmc6.jpg]

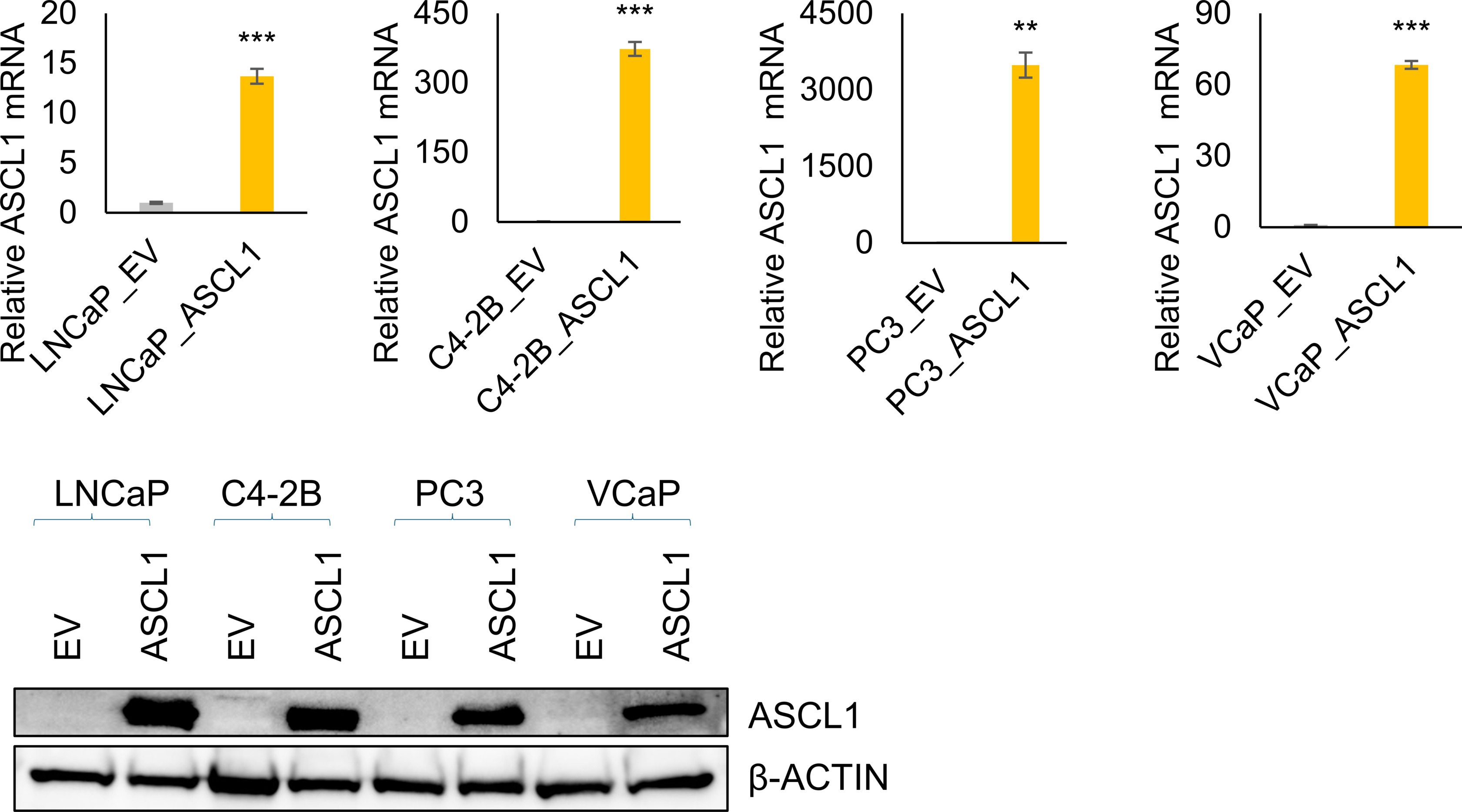

Supplement: Supplementary file 7 [file mmc7.jpg]
