# Supplementary material for: A MYC family switch: L-MYC drives and maintains neuroendocrine lineage programs in prostate cancer
Source: Neoplasia. 2026 Apr 17;77:101307. doi: 10.1016/j.neo.2026.101307 (PMC13098336; doi:10.1016/j.neo.2026.101307)
Supplement: Supplementary file 8 [file mmc8.docx]

**Supplementary Figure Legends**

**Supplementary Figure 1**

Single-cell RNA-sequencing analysis showing MYCL expression across prostate epithelial and stromal cell populations. Elevated MYCL expression is observed in NEPC cells and AdPC_ARhi populations compared with other adenocarcinoma and non-epithelial cell types.

**Supplementary Figure 2**

Heatmaps showing relative gene expression of neuroendocrine markers (ASCL1, INSM1, CHGA), AR-associated luminal markers (KLK3, NKX3-1, SPDEF), YAP1, and MYC family genes (MYC, MYCL, MYCN) across multiple prostate cancer cohorts, including Beltran, SU2C, Labrecque mCRPC, WCDT, Prostate Atlas, LuCaP patient-derived xenograft, and CTPC datasets. Gene expression values are shown as log₂(TPM + 1). Samples are grouped according to pathological or molecular lineage classifications previously described in their respective studies. Heatmap colors indicate relative expression levels (blue = low, white = intermediate, red = high).

**Supplementary Figure 3**

Scatter plots showing Pearson correlations between MYCL or MYC expression and neuroendocrine markers (ASCL1, INSM1, CHGA) across multiple prostate cancer datasets, including Beltran, SU2C, Labrecque mCRPC, WCDT, Prostate Atlas, LuCaP, and CTPC. Gene expression values are shown as log₂(TPM + 1). Each point represents an individual sample colored by molecular subtype. Linear regression lines are shown with Pearson correlation coefficients and corresponding *P* values. MYCL shows positive correlations with neuroendocrine markers, whereas MYC shows negative correlations.

**Supplementary Figure 4**

(A) MYCL expression validation. Relative MYCL mRNA expression measured by RT–qPCR confirms successful MYCL overexpression in C4-2B, LNCaP, and PC3 cells compared with control (Ctrl) cells. Immunoblot analysis further validates MYCL protein expression, with β-ACTIN serving as a loading control.

(B) Left: Cell proliferation was measured by trypan blue exclusion-based cell counting and quantified by area under the curve (AUC) analysis, demonstrating a mild reduction in proliferative capacity in MYCL-overexpressing cells compared to control. Right: Heatmap of differentially expressed genes (|log₂FC| ≥ 0.5, FDR < 0.05) reveals enrichment of cell cycle regulatory genes, consistent with reduced proliferation upon MYCL expression.

(C) Immunoblot analysis validated MYCL protein overexpression in cells co-transfected with EV or MYCL and an AR promoter-driven luciferase reporter, with β-actin serving as a loading control.

**Supplementary Figure 5**

(A) Scatter plot showing the relationship between MYCL copy number and MYCL mRNA expression. Each point represents an individual tumor sample. MYCL copy number is shown as log₂ copy-number values, and MYCL mRNA expression levels (log₂ normalized expression values) are shown on the y-axis. Spearman correlation analysis was performed to assess the association between MYCL copy number and MYCL transcript levels, with the correlation coefficient (r) and corresponding *P* value indicated in the plot.

(B) Scatter plot showing the relationship between MYCL mRNA expression and copy number variation (CNV) across prostate cancer cell lines from the CCLE dataset. Gene expression values are shown as log₂(RPKM + 1).

**Supplementary Figure 6**

(A) Box plot comparing L-MYC expression (log₂(TPM + 1)) between non–small cell lung cancer (NSCLC; n = 46) and small cell lung cancer (SCLC; n = 42) samples, demonstrating significantly higher L-MYC expression in SCLC (p<0.05).

(B) Heatmap of normalized gene expression showing lineage markers and MYC family genes across NSCLC and SCLC samples. Neuroendocrine markers (ASCL1, NEUROD1, INSM1, CHGA) and MYCL are enriched in SCLC, whereas MYC and YAP1 are relatively enriched in NSCLC. Gene expression values are shown as log₂(TPM + 1), with heatmap colors indicating relative expression levels (blue = low, white = intermediate, red = high).

(C) Correlation scatter plots showing relationships between MYC family genes (MYCL, MYC, MYCN) and neuroendocrine markers (ASCL1, INSM1, CHGA) across NSCLC and SCLC samples. Gene expression values are shown as log₂(TPM + 1). Each point represents a tumor sample colored by pathology. Black regression lines indicate linear fits, with Pearson correlation coefficients (r) and corresponding *P* values shown in each panel.

(D) Box plots showing MYC and MYCL expression (log₂(TPM + 1)) following ASCL1 knockdown (KD) compared with control in SCLC cell lines DMS53, H209, and H2107. Results suggest an association between ASCL1 and MYCL expression across models. Statistical significance was determined using DESeq2-adjusted *P* values (padj; ns, not significant; padj < 0.05).

**Supplementary Figure 7**

Quantification of ASCL1 mRNA expression by qRT-PCR in prostate cancer cell lines (LNCaP, C4-2B, PC3, and VCaP) following ASCL1 overexpression. ASCL1 expression is significantly elevated compared to empty vector (EV) controls. Immunoblot analysis confirms ASCL1 protein overexpression in the indicated cell lines, with β-ACTIN used as a loading control.
