# Supplementary material for: A MYC family switch: L-MYC drives and maintains neuroendocrine lineage programs in prostate cancer
Source: Neoplasia. 2026 Apr 17;77:101307. doi: 10.1016/j.neo.2026.101307 (PMC13098336; doi:10.1016/j.neo.2026.101307)
Supplement: Supplementary file 9 [file mmc9.pptx]

## Slide 1
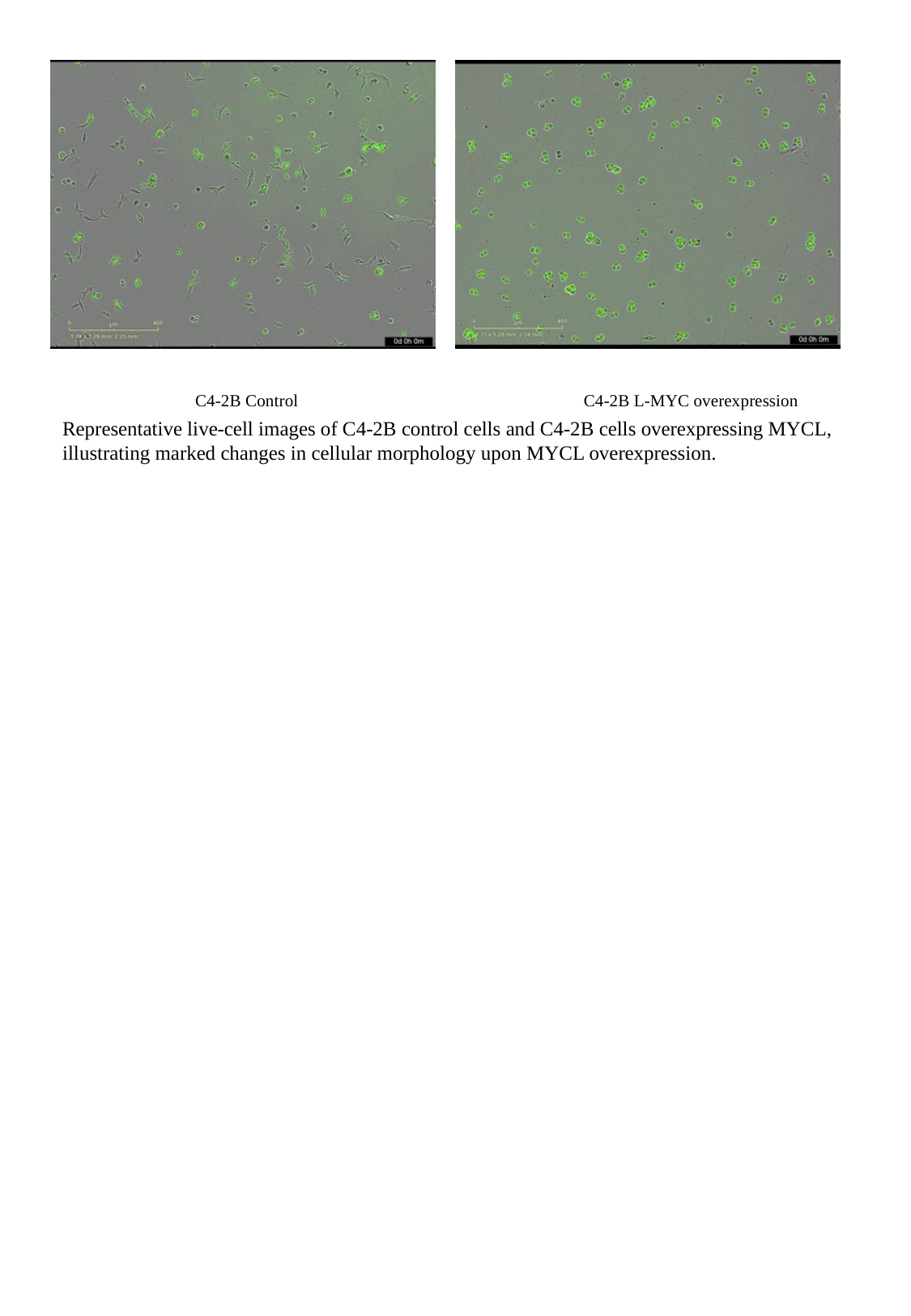

C4-2B L-MYC overexpression
C4-2B Control
Representative live-cell images of C4-2B control cells and C4-2B cells overexpressing MYCL, illustrating marked changes in cellular morphology upon MYCL overexpression.
